# Transgenic Mouse Models to Study the Development and Maintenance of the Adrenal Cortex

**DOI:** 10.3390/ijms232214388

**Published:** 2022-11-19

**Authors:** Nour Abou Nader, Gustavo Zamberlam, Alexandre Boyer

**Affiliations:** Centre de Recherche en Reproduction et Fertilité, Faculté de Médecine Vétérinaire, Université de Montréal, Saint-Hyacinthe, QC J2S 7C6, Canada

**Keywords:** transgenic mice, adrenal cortex, development, maintenance

## Abstract

The cortex of the adrenal gland is organized into concentric zones that produce distinct steroid hormones essential for body homeostasis in mammals. Mechanisms leading to the development, zonation and maintenance of the adrenal cortex are complex and have been studied since the 1800s. However, the advent of genetic manipulation and transgenic mouse models over the past 30 years has revolutionized our understanding of these mechanisms. This review lists and details the distinct Cre recombinase mouse strains available to study the adrenal cortex, and the remarkable progress total and conditional knockout mouse models have enabled us to make in our understanding of the molecular mechanisms regulating the development and maintenance of the adrenal cortex.

## 1. Introduction

The adrenal gland is an organ formed of two main regions: the centrally located medulla that produces catecholamines, and the adrenal cortex that produces steroid hormones essential for body homeostasis in mammals. The adrenal cortex is further organized into concentric zones that produce distinct steroid hormones. The outermost zone of the adrenal cortex, the zona glomerulosa (zG), secretes aldosterone. The intermediate zone, the zona fasciculata (zF), secretes corticosterone or cortisol depending on the species. Finally, the inner zone that is absent in rodents, the zona reticularis (zR), secretes dehydroepiandrosterone and dehydroepiandrosterone sulfate (DHEA/DHEAS).

The development and maintenance of the mammalian adrenal cortex is complex and includes five main sequential steps: (1) formation of a common primordium with the gonads known as the adrenogonadal primordium (AGP); (2) separation of the AGP into the gonadal primordium (GP) and the adrenal primordium (AP), with the latter being responsible for the formation of the fetal adrenal cortex; (3) encapsulation of the fetal adrenal cortex, following the invasion of neural crest-derived cells that will form the future chromaffin cells of the medulla; (4) replacement of the fetal cortex by the definitive adrenal cortex; and (5) establishment of zonation (zG, zF and zR) and subsequent maintenance of these zones.

Foundations for the study of the adrenal cortex were laid 139 years ago, when it was first suggested that cells forming all three of the above-mentioned adrenocortical zones originate from the outer capsule and migrate inwards to ultimately die at the boundary between the adrenal cortex and the medulla [1]. Although this theory was reinforced five decades later by the study of cellular renewal in adrenal injury models [2,3], it took transgenic mouse technology to begin to understand the molecular mechanisms regulating the development and maintenance of the adrenal cortex. The objectives of the present review are: (1) to detail the mouse Cre strains available to study adrenal cortex development and maintenance; and (2) to present the main findings acquired from these different models, from AGP formation up to the postnatal maintenance of the adrenal cortex.

## 2. Mouse Models Used to Study the Adrenal Cortex Development and Maintenance

In the last three decades, gene targeting approaches have been the most insightful technique to comprehend the mechanisms regulating the development and maintenance of the adrenal cortex. Aside from traditional total knockout (KO) mouse models, several Cre recombinase mouse strains have been generated and used to perform fate mapping studies and to conditionally (cKO) inactivate genes of interest in the adrenal cortex (Table 1, Supplementary Table S1). Before presenting the results obtained with these mouse models, it is important to first present the specificities and the limitations of the Cre recombinase mouse strains models used in these studies, to better understand the relevance of the results acquired from them so far.

### 2.1. Mouse Strains to Study AGP Formation

Adrenal development is first initiated with the thickening of the coelomic epithelium, followed by subsequent delamination of a group of cells that migrate inwards to form the AGP beginning at around e9.5. Most information obtained for this stage of development comes from studies employing KO animals. Identifying the best promoter to drive Cre expression and generate cKO to specifically study this step of development have proven difficult because genes expressed in the coelomic epithelium are also expressed in several other tissues. For example, *Gata4* (gene names/abbreviations are listed at the end of this review) and *Wt1* are two key transcription factors expressed in the coelomic epithelium and involved in the early steps of AGP formation [5,61]. However, both genes present broad expression, including in the developing heart, and using them to drive a Cre recombinase and generate cKO could lead to the death of the embryo at a time point that precedes the thickening of the coelomic epithelium. *Gata4*-Cre [62,63] and *Wt1-Cre* [12,64,65] strains have therefore not been employed to generate cKO models and evaluate early stages of adrenal development. Tamoxifen inducible models have provided a more promising solution to understand this stage of development, as demonstrated by the study of the role of GATA4 in the coelomic epithelium [5]. In this study, three mouse strains were used to inactivate *Gata4* at e8.75: the *CAG-CreER* [4]; the *Osr1^eGFP-CreERt2^* [7]; and the *Wt1^CreERt2^* [12] (mouse strains expressing, respectively, Cre following tamoxifen injections ubiquitously [4] or in the intermediate mesoderm/early coelomic epithelium [7,8,12,66,67,68] among other tissues). It was further suggested that creating mutants with both *Osr1-* and *Wt1*-driven Cre alleles in the same animal might be the best solution to study the earliest steps of the AGP formation [5], since recombination with both *Osr1^eGFP-CreERt2/+^* and *Wt1^CreERt2/+^* strains has shown variant efficiency while the *CAG-CreER* strain increases the risk of an indirect effect [5]. Two other mouse strains have been generated that could be useful to evaluate the AGP. First, the *Tbx18^Cre^* mouse strain targets, among other tissues, the adrenal precursors in the anterior coelomic epithelium [9,10]. This mouse strain has only been used once to evaluate the AGP [10]. Finally, the generation of a *Gata4^CreERt2^* strain was recently reported [11]. However, this strain has not yet been used to study the development of the AGP.

### 2.2. Mouse Strains Using Nr5a1 Regulatory Sequences to Drive Cre Expression

Concomitantly to coelomic epithelium thickening and delamination, *Nr5a1* expression rapidly increases in the forming AGP. Contrary to *Gata4* and *Wt1, Nr5a1* expression is maintained in the AP, and the fact that its expression is mainly restricted to endocrine/steroidogenic tissues, makes its regulatory region an interesting driver of Cre expression. Indeed, two *Nr5a1*-*Cre* mouse strains have been generated [14,30] and, to date, they remain the most common strains used to study the development and maintenance of the adrenal glands. A less efficient version of one of these *Nr5a1*-*Cre* strains called *Nr5a1*-*Cre^low^* (in opposition to *Nr5a1*-*Cre^high^*) [14] has also been reported, but has only been used seldomly since its driven recombination occurs in fewer cells. Several aspects must be considered when analyzing cKO models generated using the *Nr5a1*-*Cre* strains. First, *Nr5a1* is expressed in the AGP, the fetal cortex and the adult cortex, making it difficult to determine if a phenotype observed at a certain time point indicates a role for the deleted floxed target genes at this particular time point, or if the observed phenotype is actually associated with an alteration that has started in a previous step of development. For example, the fetal cortex initially contributes to adult cortex formation [35,36] and inactivating a gene important for the development of the fetal cortex could also indirectly affect the formation of the adult cortex. Secondly, conditional deletion of the gene of interest will also be performed in other steroidogenic and endocrine cells including the Leydig and Sertoli cells in the testis, the granulosa and theca cells in the ovary, the gonadotropes in the pituitary and the neurons of the ventromedial hypothalamus [14,30]. Considering the main hormones produced by each of these cells, it is likely that the loss of the target gene expression in any of these cells might affect the maintenance of the adrenal cortex indirectly. Androgens [37,54] and, to a lesser degree, estrogen [69] have been shown to affect homeostasis of the adrenal cortex, while luteinizing hormone (LH) has been shown to induce the transdifferentiation of adrenal hyperplastic spindle-shaped cells into sex-steroid producing cells in gonadectomized mice [70,71]. Furthermore, it was demonstrated that lesions of the ventromedial hypothalamus in rats increased the adrenal weight and inhibited corticosterone/basal adrenocorticotropic hormone (ACTH) diurnal rhythm feedback [72]. Gonadal and gonadotropic hormones (and potentially corticosterone circadian rhythm) should therefore be evaluated, to determine if an observed abnormal phenotype following gene inactivation is exclusively due to alterations happening in the adrenal cortex and/or depends on hormones secreted from other tissues. Finally, it was recently demonstrated that conditional deletion of the genes of interest could also occur in a subset of dermal fibroblast progenitors when using the *Nr5a1*-*Cre^high^* model [73]. Although inactivation of a gene of interest in these cells is unlikely to indirectly affect the adrenal cortex, this expression must be considered when characterizing the phenotype of mouse models. This is particularly true for mouse models that attempt to simulate a complex syndrome affecting multiple organs like Carney complex [73].

Three other mouse strains using *Nr5a1* regulatory sequences to drive Cre expression have been reported. First, a tamoxifen inducible *Nr5a1^eGFP-CreERt2^* mouse strain was created by the Welcome Trust Sanger Institute. That strain, which has not yet been used, could technically target *Nr5a1*+ cells at specific time points to only study postnatal steroidogenic cells, for example. Secondly, two strains, the *FAdE/Nr5a1-Cre* and the *FAdE/Nr5a1-CreERT2,* have been reported [35]. These strains use the enhancer that selectively drives *Nr5a1* expression in the fetal adrenal cortex to specifically express Cre in the fetal adrenal cortex [35]. These two models have been used mainly to perform lineage experiments and determine the fate of the fetal adrenal cortex [35,36]. However, the *FAdE/Nr5a1-CreERT2* was used once for gene inactivation [37]. Interestingly, the *FAdE/Nr5a1-CreERT2* model can specifically target the fetal adrenal cortex without subsequently affecting the definitive cortex if the recombinase Cre is activated after e14.5 [35].

### 2.3. Mouse Strains Using Regulatory Sequence of Genes Coding for Steroidogenic Enzymes to Drive Cre Expression

Three strains using the promoter of the steroidogenic enzyme *Cyp11a1* to drive Cre expression have also been employed to inactivate genes in the adrenal cortex. The first two of these models used either 4.4Kb of the human *CYP11A1* promoter [38] or 2.8Kb of the mouse *Cyp11a1* promoter [40]. In both strains, Cre expression is detected in the fetal and adult adrenal cortex and in Leydig cells. However, Cre expression is also detected in the theca cells and corpus luteum of the postnatal ovaries [38,40] and, for the human *Cyp11a1-iCre* strain, at lower levels in the female gonads and in the diencephalon and midbrain [38]. Expression in the brain was not evaluated in the mouse *Cyp11a1-iCre* model [40]. More recently, a third model (called *Cyp11a1^Gfp,Cre^*) was generated to drive Cre expression under the endogenous *Cyp11a1* promoter [42]. In this model, the integration of a GFP/Cre cassette was used to disrupt the *Cyp11a1* exon containing the *ATG* site [42]. Again, Cre expression was detected in the fetal and adult testis, adrenal cortex and adult ovary (as well as in the cerebellum) [42]. Although these models are a little bit more specific than the *Nr5a1*-*Cre* strains, similar problems will arise since sexual hormones will/could also be affected in them.

Other strains have been generated/used to target recombination in the adrenal cortex in a more specific matter. First, a *Cyp11b2^Cre^* knocking allele (better known as AS*^Cre^*) has been created to target zG cells. Using tracing experiments, it was demonstrated that a few zG cells were marked between e16.5 and 1dpp with all zG cells marked at 6 weeks after birth [48]. Tracing experiments further demonstrated that all cells of the adrenal cortex were eventually marked due to centripetal migration and lineage conversion of zG cells into zF cells [48]. Although the AS*^Cre^* is specific to the adrenal cortex and has the potential to inactivate a gene of interest in all the zones of the adrenal cortex, it was demonstrated that the zF cell population can be maintained independently of the zG cell population when the capacity of zG cells to differentiate into zF cells is affected [48]. This finding suggests that this strain might not be useful to study the function of a gene in both the zF and zG when genes essential for zG cell survival or differentiation are inactivated [48,50]. Furthermore, loss of zG cells or zG functions can potentially lead to an increase in the proliferation of the non-recombined progenitor and stem cell populations, and their subsequent differentiation into zG steroidogenic cells leading to a mosaic of recombined steroidogenic zG cells and consequently to a weaker phenotype. Similarly, lineage conversion of zG cells into zF cells could also be accelerated if the deleted gene is particularly important for zF functions as the tissue tries to replace/replenish the zF with functional cells. Cells might therefore spend insufficient time in their zG state to allow efficient recombination of some floxed alleles before their transdifferentiation into zF cells. This could also be exacerbated in females in which complete adrenal cortex turnover is three-times faster than in males [54]. More recently, a strain in which a P2A-eGFP-Cre cassette was integrated before the 3′ UTR region of the *Cyp11b1* gene, was generated using Crispr technology to specifically target the zF. However, to date, only one scientific paper (written in Chinese) has been published using this model [51].

### 2.4. Other Mouse Strains Used to Target the Adrenal Cortex

Other strains have been generated/used to target recombination in the adrenal cortex. In the *Akr1b7-Cre* strain, recombination is observed in about 80% of the adult adrenocortical cells [45]. Recombination is also observed in the adrenal cortex starting at e14.5 and persists in the presumptive X-zone cells until 10dpp [45]. This suggests that Cre recombination occurs in both the fetal and definitive adrenocortical cells. As by e14.5, the fetal adrenocortical cells do not contribute anymore to the formation of the adult cortex [35]; this also suggests that an abnormal phenotype observed postnatally will directly come from the inactivation of the gene of interest in the definitive cortex. Interestingly, Cre activity is not detected in the gonads (though recombination can be observed in some structures of the kidney) facilitating the interpretation of the phenotype [45].

The *Gli1 ^CreERt2^* strain [52] targets, among other tissues, the capsular adrenal stem cells. This strain has been used for both tracing experiments and for gene inactivation. However, it is important to note that while the capsular stem cells contribute to the adrenocortical steroidogenic cell lineage in juvenile males and females, their contribution is limited to the females in adult mice [53,54]. The dimorphic contribution of the stem cell population and the timing of the inactivation should therefore be accounted for when this model is used. The importance of the Hedgehog signaling in numerous tissues limits the potential usage of this strain. Similarly, a few other mouse strains targeting different cell populations in the adrenal cortex have mostly been used for tracing experiment due to concomitant recombination in several tissues. These strains, respectively, target a subpopulation of capsular cells (the previously mentioned *Wt1^CreERt2^* [12]), the subcapsular progenitor cells (*Shh^Cre^* and *Shh^CreERt2^* [56]), all zG cells (*Axin2^CreERt2^* [57], *Wnt4 ^CreERt2^* [58]) or a stress induced adrenocortical progenitor cell population (*Nes-*CreERt2) [59,60].

## 3. AGP Development

**Figure 1:**
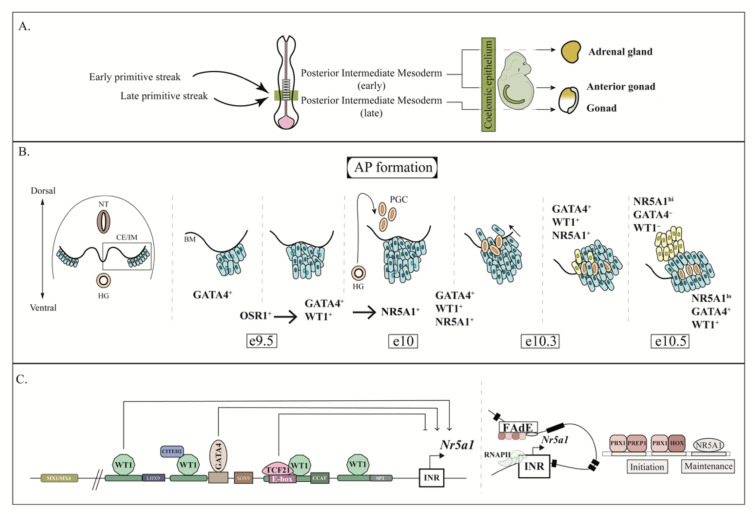
Key events in for the early stages of the development of the fetal adrenal cortex in mice: (**A**) Schematic model for the anterior–posterior regionalization of the posterior intermediate mesoderm, the coelomic epithelium and the AGP in mouse. Fate mapping experiments suggest that cells from the early (anterior) primitive streak migrate to form the early posterior intermediate mesoderm, which is followed by subsequent formation of both the adrenal and the gonad, while cells from the late (posterior) primitive streak migrate to form the late posterior intermediate mesoderm, which is followed by formation of the gonad but not the adrenal gland. (**B**) Schematic model for the development of the AP in mouse. The schematic representation of a transverse view of an embryo shows that the AGP arises from the thickening and delamination of the coelomic epithelium, a process that is initiated around 9.5 and that necessitates the contribution of GATA4 OSR1, WT1 and NR5A1. Once AGP is formed it is invaded by the PGCs (around e10.0), which leads to the separation of the AP from the GP (at e10.5 when a population of cells expressing high levels of NR5A1 begins to migrate dorsomedially). (**C**) Overview of the regulation of the transcription of *Nr5a1* during AGP and AP formation. In the coelomic epithelium/AGP, *Nr5a1* expression is initiated by the binding of several factors to its proximal promoter (only the transcription factors relevant to adrenal development are depicted). Elevated expression of *Nr5a1* in the AP is initiated by the binding of PREP1, PBX1 and HOXs to the FadE enhancer located in the exon 4. Elevated expression of *Nr5a1* in the AP is further maintained by an autoregulatory loop. AGP = adrenogonadal primordium, AP = adrenal primordium, BM = basement membrane. CE/IM = coelomic epithelium/intermediate mesoderm, FadE = fetal adrenal enhancer, GP = gonadal primordium, HG = hindgut, PGCs = primordial germ cells, NT = neural tube.

As previously mentioned, the adrenal cortex and the gonads arise from the thickening of the coelomic epithelium. However, the genesis of these organs is initiated at an earlier time point of embryonic development. Indeed, recent fate mapping studies have demonstrated that the coelomic epithelium could derive from the posterior intermediate mesoderm which emerges from the primitive streak [8,74]. In mice, it was demonstrated that mesenchymal cells originating from the early primitive streak (and subsequent early posterior intermediate mesoderm/coelomic epithelium) contribute to both the adrenal and the anterior gonad formation, while cells emerging from the late primitive streak (and subsequent late posterior intermediate mesoderm/coelomic epithelium) contribute solely to the gonad formation [8] (Figure 1A). In humans and monkeys the adrenal and gonad arise from two distinct regions of the coelomic epithelium (anterior and posterior regions, respectively) [74], suggesting that complete segregation of both tissues arises earlier in these species. Furthermore, it was suggested that a *Hox* gene code is involved in the anterior/posterior regionalization of the coelomic epithelium [8,74]. Finally, in the chicken, the adrenal gland seems to arise from the inner layer of the coelomic epithelium while the gonad arises from the outer layer [75]. This regionalization of the coelomic epithelium could explain the discrepancies observed following the inactivation of genes considered critical for the thickening of the coelomic epithelium and AGP formation. Indeed, while inactivation of *Osr1* [76] or *Wt1* [61,77] leads to adrenal and gonadal agenesis (Table 2, list of mouse models), inactivation of *Emx2* or *Lhx9* leads to agenesis of the gonads without affecting adrenal development [78,79,80].

Three genes, *Gata4* and the aforementioned *Osr1* and *Wt1,* appear to play a central role for AGP thickening (Figure 1B). *Gata4* is detected as early as e8.0 in the coelomic epithelium [112]. Global inactivation of *Gata4* leads to embryonic death before e9.0 [113,114] and cannot be used to study AGP formation. However, its conditional inactivation in the coelomic epithelium at e8.75 completely abolishes its thickening, as well as the subsequent fragmentation of the basement membrane underneath the coelomic epithelium and the proliferation and delamination of the epithelial cells at e10.3 [5]. *Osr1* is also first expressed in the coelomic epithelium/mesenchyme at e8.0-8.5 (66,74,76), and as previously mentioned, its inactivation leads to complete agenesis of the gonads and adrenal glands [76]. Similarly, *Wt1* is also detected around e9.0 in the coelomic epithelium [68,115], and its inactivation also leads to complete agenesis of the gonads and adrenal glands [61,77].

The study evaluating the function of GATA4 in the coelomic epithelium further suggested that GATA4 initiates the thickening process, while OSR1 and WT1 were not essential for the initiation of AGP formation (as a small thickening of the coelomic epithelium was initially observed in *Osr1*^−/−^ [5] and *Wt1^−/−^* animals [61]). However, it is important to note that the functional hierarchy of these genes in the process of AGP formation has not been thoroughly evaluated. *Wt1* appears to be a target of OSR1, at least in some tissues [76], but this was not clearly demonstrated in the AGP. It was also demonstrated that GATA family members can regulate *Wt1* expression in Jurkat and K562 cell lines by binding to a 3′ enhancer [116]. However, *Wt1* does not appear to be a target of GATA4 in the AGP [5]. GATA4 also does not appear to be a target of WT1 in the AGP [117]. On the other hand, WT1 and GATA4 have been shown to act in synergy to promote the transcription of genes important for sex determination/differentiation [118], suggesting that they could act in synergy in the AGP. More recently, the ontogenic ancestries of the AGP in mouse, human and monkey were evaluated [8,74]. Interestingly, in human and monkey *WT1* expression precedes *GATA4* expression in the AGP [8,74]. Furthermore, it appears that the adrenal primordium specifically originates from a portion of the anterior coelomic epithelium that does not express *GATA4* [74]. Although it was also suggested in these experiments that *Wt1* expression precedes *Gata4* expression in the mouse, the adrenocortical and gonadal lineages (and *Wt1*/*Gata4* expression) are initially joint in the coelomic epithelium [8,74].

No matter which gene is expressed first, inactivation of *Gata4* and *Wt1* both lead to a decrease in the expression of *Nr5a1* [5,83], and *Nr5a1* inactivation leads to adrenal (and gonadal) agenesis [94] suggesting that *Nr5a1* acts downstream of these factors for AGP formation. It was demonstrated in vitro that both these factors can directly regulate *Nr5a1* transcription by binding to its proximal promoter. WT1 have been shown to bind to four sites located in the first 500bp of the *Nr5a1* proximal promoter (Figure 1C) [119]. Interestingly, if the mutations of all four WT1 binding sites induce an important decrease in *Nr5a1* promoter activity [83], the introduction of a point mutation in any single WT1 binding site rather increases *Nr5a1* proximal promoter activity. This suggests that WT1 can potentially both activate and repress *Nr5a1* activity depending on the cellular context [119]. GATA4 is also able to bind to the proximal promoter of *Nr5a1* and enhance its activity in vitro [120] (Figure 1C). This activation was achieved in Sertoli and pituitary cell lines, but not in Leydig and adrenal cell lines [120]. Again, these findings reinforce the conclusion that the cellular type/context is important to comprehend the mechanisms regulating *Nr5a1* in the AGP/adrenal cortex.

In addition to *Gata4* and *Wt1*, the inactivation of *Cited2* [82,83], *Tcf21* [36], and *Insr/Igf1r* [89] also leads to early developmental defects of the adrenal gland. Like the inactivation of *Gata4* and *Wt1*, the inactivation of *Cited2* and *Insr/Igf1r* also decreases *Nr5a1* expression [83,89]. CITED2 is first expressed in the coelomic epithelium at e10.0 [83]. Contrary to the inactivation of *Gata4* and *Wt1*, inactivation of *Cited2* has a greater impact on adrenal development than the gonad as the gonads appear to recover from early differentiation defects [83]. CITED2 physically interacts with WT1 and stimulates its transcriptional activity at the *Nr5a1* basal promoter [83]. Interestingly, *Cited2* expression remains high in the AP but decreases in the GP [83], suggesting that *Cited2* might have additional roles in the AP after its separation.

The loss of *Insr/Igf1r,* the receptors for insulin and insulin-like growth factors, leads to a ≈40% reduction of NR5A1+ cells associated with a reduction in the proliferation rate of GATA4+ cells, and an alteration of a quarter of the genes known to be involved in the development of the AGP/bipotential gonad [89]. Despite this more global effect, it is still possible that insulin growth factor (IGF) signaling regulates NR5A1 as INSR/IGF1R have been shown to activate the mitogen-activated protein kinase (MAPK) signaling pathway [121], which promotes phosphorylation-dependent-NR5A1 activation [122].

Finally, contrary to the previously mentioned genes, TCF21 represses *Nr5a1* transcriptional activity by binding to a E-BOX site [123,124] that overlaps with a WT1 binding site [119] (Figure 1C). Interestingly, it was demonstrated that inactivation of *Tcf21* did not affect AGP formation, but rather led to incomplete separation of the AP and the GP [36]. This suggests that *Nr5a1* must be tightly regulated during the development of the AGP/AP.

Other genes expressed in the AGP such as *Six1*, *Six4* and *Cbx2* are also able to regulate the transcriptional activity of *Nr5a1.* SIX1/SIX4 are able to bind and activate the transcriptional activity of *Nr5a1* proximal promoter [108], while CBX2, a component of the mammalian polycomb repressive complex-1 required for chromatin remodeling and histone modification, has been shown to bind several regions of the *Nr5a1* genomic region [81]. However, the inactivation of these genes suggests that they are more important to gonadal development than adrenal development [108,109,125,126,127] as their inactivation only leads to marginal or mild adrenal hypoplasia [81,108,109,127]. Nonetheless, these factors might fine-tune adrenal development.

## 4. AP and Fetal Adrenal Development

If GATA4 and WT1 are considered two of the main regulators for the initial formation of the AGP, their expression in mice is switched off in the AP, just after its separation from the GP occurs [13,128]. This suggests that these genes prevent the differentiation of AGP cells into the adrenal steroidogenic cell lineage. Indeed, it was demonstrated that the ectopic expression of high levels of the WT1-KTS isoform (the isoform able to bind DNA and act as a transcription factor) in NR5A1+ cells of the AGP leads to the maintenance of GATA4 expression, reduced NR5A1 expression, and the formation of abnormal small adrenal glands [13]. Furthermore, WT1 has been shown to bind the promoter of *Tcf21* suggesting that WT1 could also inhibit *Nr5a1* expression indirectly [13]. In an initial study using chimeric mice (generated by the injection of Gata4^−/−^ ES cells in blastocysts), it was also suggested that GATA4 was not essential for adrenocortical cell differentiation [129]. However, concomitant inactivation of *Gata4* and *Gata6* (but not *Gata4* alone) in NR5A1+ cells of the AGP leads to adrenal agenesis, suggesting that GATA4/6 have redundant activity in the AGP before AP separation and downregulation of GATA4 expression [21,22]. Interestingly, inactivation of *Gata6* alone in these cells affects adrenal development suggesting that GATA6 is important for later stages of adrenal development [31]. Following *Gata6* inactivation, expression of GATA4 is maintained in the AP. However, residual NR5A1+ cells did not express GATA4, which suggests that GATA4+ cells are unable to commit to the adrenal steroidogenic lineage. The maintenance of GATA4 expression in this model could be due to a compensatory mechanism. It is also possible that GATA6 normally represses *Gata4* expression in later stages of adrenal development, as it was demonstrated in H9 and P19CL6 cell lines and heart development that GATA4 and GATA6 can mutually and directly regulate their transcriptional activity [130,131,132].

As mentioned previously, *Nr5a1* expression closely follows *Gata4* and *Wt1* expression in the AGP, and global inactivation of *Nr5a1* leads to adrenal and gonadal agenesis [94]. However, contrary to GATA4 and WT1 expression, robust NR5A1 expression is maintained in the AP following its separation from the AGP. Such robust *Nr5a1* transcription is possible due to the activation of the FadE located in the intron 4 of *Nr5a1* [133]. Studies employing both cell line assays and transgenic mouse models have demonstrated that *Pbx/Prep/Hox* binding sites were necessary for the initiation of the transcription by the FadE (highlighted by the fact that deletion of *Pbx1* leads to adrenal agenesis [100]), while *Nr5a1* transcription is further maintained by a NR5A1 positive autoregulatory loop [133] (Figure 1C). It has also been suggested that NR5A1 dosage is critical for AP development, and it is thought that cells expressing higher levels of NR5A1 give rise to cells that will form the AP whereas cells expressing lower levels will form the GP. This was originally proposed because the adrenal glands of *Nr5a1*^+/−^ heterozygous animals were highly hypoplastic, while the gonads were not [95]. A subsequent study demonstrated that the loss of one *Nr5a1* allele decreased the number of adrenal precursor cells within the AGP but not the gonadal GATA4+ precursor cells [96]. Inactivation of *Nr5a1* using the m*Cyp11a1*-*Cre* model also demonstrated that Nr5a1- cells adopt a more elongated and flat shape reminiscent of less differentiated cells, but GATA4 and WT1 expression were not evaluated in this model [40]. Furthermore, overexpression of NR5A1 (using a basal *Nr5a1* promoter and the FadE to drive its expression) led to ectopic adrenal tissue formation in the thorax [97], again suggesting that high NR5A1 expression is necessary for the proper differentiation of the AP. Interestingly, separation of the AP and GP was also affected in this model, suggesting that *Nr5a1* dosage is important for this process [97]. As previously mentioned, TCF21 is also important for the separation of the AP and GP and negatively regulates *Nr5a1* expression (36,123,124), suggesting that TCF21 could be essential for *Nr5a1* dosage.

Aside from NR5A1, it was also demonstrated that FGFR2 is important for AP formation. *Fgfr2* inactivation also leads to major adrenal hypoplasia [10,20]. Serial section and 3D reconstruction analyses revealed that the number of NR5A1+ cells was initially normal in AP of e10.5 mutant animals, but that a two-fold reduction was observed at e11.5. It was further demonstrated that cell proliferation of NR5A1+ cells was also reduced by around 50% at e11.5 and e12.5, and that apoptosis increased at e12.5 suggesting that FGFR2 is required for the expansion of the AP by regulating both cell proliferation and apoptosis [10].

As previously mentioned, the late stages of fetal adrenal development are difficult to evaluate as the inactivation of the gene of interest will occur in cells of both the fetal and adult cortex using most Cre strains, and both cortices will be present at these later time points. An indirect manner to confirm the importance of factors involved in later stages of the fetal cortex development would be to evaluate the fetal cortex at birth, which is referred as the X-zone (before its eventual regression at puberty in males or after the first pregnancy in females [134,135]). It could be expected that inactivation of genes that normally regulate the fetal cortex development positively would lead to the absence or a decrease in the size of the X-zone at birth; while inactivation of genes that normally regulate the fetal cortex development negatively would lead to the opposite effect and the presence of a larger X-zone at birth. To illustrate this, a smaller X-zone is observed in animals with *Pbx1* haploinsufficiency [101]. As previously mentioned, *Pbx1* is a gene important for *Nr5a1* transcription from the FadE. On the other hand, *Nr0b1* knockout male mouse present a larger X-zone in young animals and X-zone regression is delayed [92]. A similar phenotype is also observed in animals with a SUMOylation deficient form of NR5A1 [92]. It was further demonstrated that SUMOylation of NR5A1 facilitates the recruitment of NR0B1 to the FadE of *Nr5a1* to inhibit its transcriptional activity [92]. Inactivation of *Gata6*, inactivation of *Siah1a* and overexpression of *Hoxb9*, respectively, lead to animals lacking an X-zone [31], having a smaller X-zone [106] and having a larger X-zone [41]. This suggests that these genes are also involved in the development of the fetal cortex.

**Figure 2:**
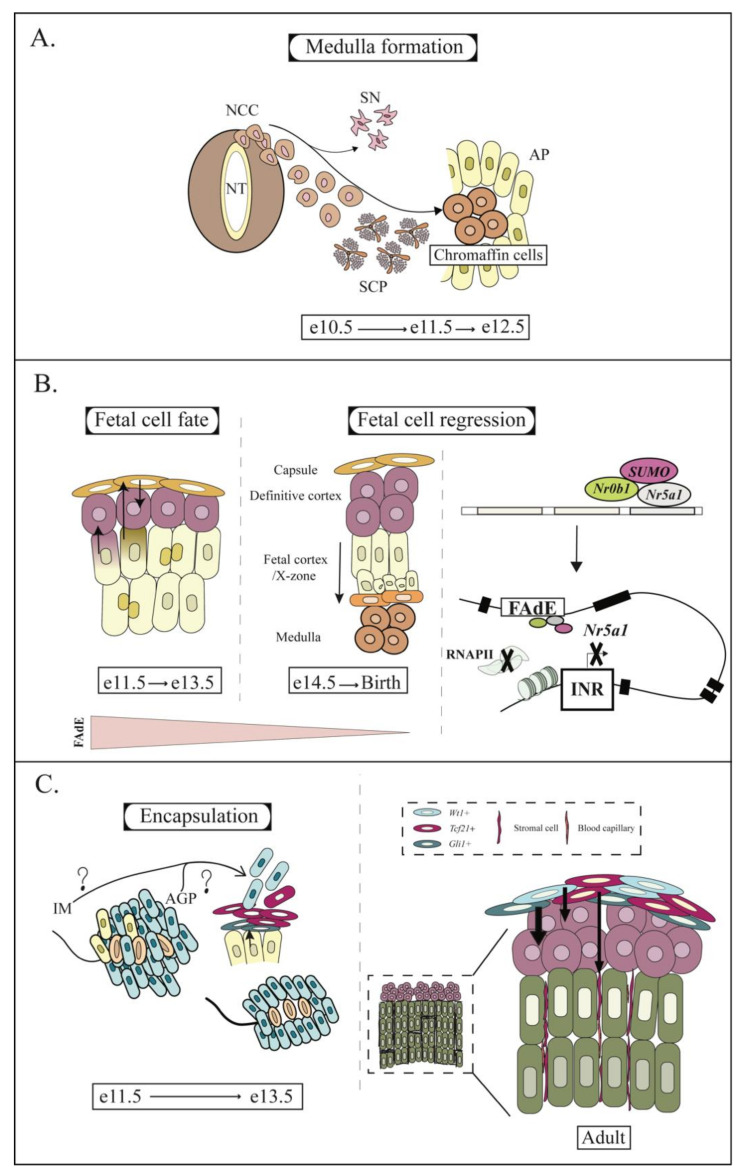
Key events in the late stages of fetal adrenal gland development and fate of the fetal adrenal cortex in mice: (**A**) Schematic model of medulla formation. Chromaffin cell differentiation requires the migration of a subpopulation of neural crest cells and their progressive differentiation into Schwann cell precursors and chromaffin cells. (**B**) Schematic model depicting the fate of the fetal adrenocortical cells. Before e14.5, adrenocortical fetal cells can differentiate into adrenocortical cells of the definitive cortex by first differentiating into capsular stem cells (or potentially by directly differentiating into cells of the definitive cortex), or they can proliferate and maintain the AP. After e14.5, cells from the AP do not contribute anymore to the definitive cortex formation. The progressive inactivation of *Nr5a1* expression via NR5A1 SUMOylation and recruitment of NR0B1 to the FAdE leads to the regression of the fetal cortex/X-zone. (**C**) Schematics model for AP encapsulation. AP encapsulation requires the contribution of cells originating from both the fetal adrenal cortex and cells potentially originating directly from the AGP or the surrounding intermediate mesoderm. Different cell subpopulations have been identified including: *Gli+* cells that are located the nearest to the adrenal cortex and that will eventually differentiate into steroidogenic cells of the definitive adrenal cortex; *Wt1*+ cells that have a more limited potential to differentiate into steroidogenic cells; and *Tcf21*+ cells that will differentiate into stromal cells of the definitive adrenal cortex. NCC = neural crest cells, NT = neural tube, SN = sympathetic neurons, SCP = Schwann cell precursors.

Soon after AP separation, a population of peripheral glial stem cells derived from the neural crest will migrate, invade the medulla, and eventually differentiate into chromaffin cells (Figure 2A). Single cell RNAseq experiments suggest that this migrating population of stem cells differentiate into sympathetic neurons (SN) and Schwann cell precursors (SCP), with the SCPs further differentiating into chromaffin cells in the AP [136,137] (although the transition between cell fates in human appears to occur in a different order [138]). Chromaffin cells are not necessary for the development of the adrenal cortex, but their presence is necessary for its proper organization [139].

## 5. Fate of the Fetal Cortex

As previously mentioned, SUMOylation of NR5A1 followed by NR0B1 binding on the FAdE will eventually inactivate *Nr5a1* transcription in the fetal cortical cells [92] (Figure 2B). This process will eventually lead to the complete regression of the fetal zone that will arise at puberty in male because of androgen action [44,140], or following the first pregnancy in female (or in old virgin females) [141,142] by an unknown mechanism (though it was suggested that progesterone might be involved in this process [143] and that androgen is probably not [43]). However, although some adrenocortical fetal cells follow this above-mentioned path, it does not appear to be the case for all of them. Indeed, tracing experiments using the FAdE driving LacZ demonstrated that if LacZ expression clearly diminished in the outside cortex (where the definitive cortex appears as discussed below) and persisted in the inner fetal adrenal cortex, some cells between the two zones appear to have transient LacZ expression suggesting that cells of the fetal cortex could differentiate into cells of the definitive cortex [35,133]. A subsequent experiment (in which the FAdE was used to drive Cre recombinase in ROSA26-LacZ reporter mice to permanently mark the adrenocortical cells) confirmed that this was indeed the case [35]. Furthermore, a study using a tamoxifen inducible *FAdE-CreERt2,* demonstrated that fetal adrenal cortical cells from e11.5 animals can differentiate into adrenocortical cells of the definitive cortex, but that their potentiality is lost at e14.5 [35]. An additional study further suggested that some adrenocortical fetal cells migrate into the developing capsule and form supporting mesenchymal cells and potential stem cells for the definitive cortex [36] (Figure 2B).

If tracing experiments suggest that the fetal adrenocortical cells can differentiate into cells of the definitive cortex, a recent single cell RNAseq analysis rather suggests that three different cell clusters (adrenal primordium, fetal zone and definitive zone) can be observed in the developing adrenal cortex [137]. Briefly, this study suggests that a subset of cells forming the adrenal primordium cluster first differentiate into cells of the fetal cortex cluster while another subset of cells differentiate into cells of the definitive cortex, suggesting a mutual exclusion of the differential potential of the adrenal primordium cluster across the other two clusters [137]. It is however important to point out that these single cell analyses were performed at time points ranging from e13.5 to P5, a time window that could be considered too late to properly observe the differentiation of the fetal adrenocortical cells into cells of the definitive cortex, as it was previously demonstrated that fetal adrenocortical cells progressively lose their capacity to differentiate into definitive adrenocortical cells between e11.5 and e13.5 before losing this potentiality at e14.5 [35].

## 6. Encapsulation of the Adrenal Cortex

Following the invasion of the fetal cortex by the chromaffin cells and concomitantly with the beginning of the transition of the fetal cortex/definitive cortex, mesenchymal-like cells will encase the forming adrenal gland and form the capsule; a process fully completed at around e13.5–e14.5. The lineage of these mesenchymal cells is not completely understood, but partially overlapping cell populations have been identified in the capsule (Figure 2C). First, the majority of the capsular cells express NR2F2 and tracing experiments demonstrate that some of these cells originate from the fetal cortex [36]. Three other cell populations have been identified as a *Gli1*+ cell population, a *Tcf21*+ cell population and a *Wt1*+ cell population. The NR2F2+ and *Gli1+* cells partially overlapped, and tracing experiments also suggest that some of the *Gli1*+ cells originate from the fetal cortex [36]. *Tcf21*+ cells do not arise from the fetal cortex but might arise from the AGP [36] or from other regions of the intermediate mesoderm (Figure 2B). Finally, a *Wt1+* cell population potentially overlaps with the *Tcf21*+ cell and with the *Gli1+* cells [13]. It was further demonstrated that WT1 could regulate the transcription of both *Tcf21* and *Gli1* [13]. Single cell RNAseq performed at e13.5 also identified *Gli1*, *Tcf21* and *Wt1* expression in the same cell clusters, while two clusters of capsular cells, *Tcf21^high^* and *Wt1^high^*, were identified in late fetal/perinatal adrenal gland suggesting that most *Tcf21*+ and *Wt1+* cells belong to distinct capsular cell populations at these time points [137]. However, it is important to note that a limited number of cells (a little over 2000 cells from whole adrenal over six different time points) were used for this latter experiment. The number of capsular cells sequenced was therefore insufficient to truly determine how many cell populations were present in the adrenal capsule [137]. Capsular cells do not express NR5A1, including the cells that originate from the fetal cortex [36] and, interestingly, NR2F2 [144], TCF21 [123,124] and WT1 [13] are all able to negatively regulate *Nr5a1* expression. This could suggest that these genes ensure that capsular cells do not express *Nr5a1*.

## 7. Development and Maintenance of the Definitive Cortex: The Key Role of Hedgehog and Canonical WNT Signaling Pathways

Fetal adrenal cortical cells can initially differentiate into adrenal cortical cells of the definitive cortex but lose this capacity after e14.5 [35]. This suggests that if the initial cells of the definitive cortex originate from the fetal cortex, the cells necessary for the late stages of the definitive cortex development (and its subsequent maintenance) have a different origin [35]. Again, tracing experiments were essential to better understand the origin of these cells. Using fate mapping it was demonstrated that capsular cells positive for *Gli1*+ (the main effector of the Hedgehog signaling pathway) were able to differentiate into steroidogenic cells of the adrenal cortex (Figure 2C), both in the embryo and postnatally, and that marked cells migrate inward while centripetally displacing older cells (27,28,36). It was further demonstrated that some capsular *Wt1+* cells were also able to differentiate into adrenocortical steroidogenic cells (Figure 2C) although the authors suggested that the *Wt1-, Gli1+* cell located in the interior side of the capsule is probably the main capsular stem cell population [13]. Contrary to the *Gli+* cells, the *Tcf21+* cells are only able to form steroidogenic cells before the formation of the capsule and only differentiate into non-steroidogenic stromal adrenocortical cells once the capsule is formed [36] (Figure 2C).

Following *Gli1+* tracing experiments, the importance of the Hedgehog signaling was further confirmed when *Shh*, which is expressed in the subcapsular cell of the adrenal cortex, was inactivated. Conditional deletion of *Shh* in the adrenal cortex using a *Nr5a1*-*Cre* strain were generated by three different groups that demonstrated that its inactivation led to adrenocortical hypoplasia [27,28,29]. This phenotype was associated with a thinning of the adrenal capsule [27,28] and a reduction in capsular cell proliferation [27], indicating that Hedgehog signaling affects capsular cells and not steroidogenic cells. This was further confirmed by the fact that the inactivation of *Smo*, a transmembrane protein essential to transduce Hedgehog signaling in the adrenal cortex, did not lead to an apparent phenotype [28]. Furthermore, the number of *Gli1+* cells was dramatically reduced in the capsule following *Shh* inactivation [28], confirming that Hedgehog signaling acts on the capsular cells. *Gli2* and *Gli3* are also important for Hedgehog signaling and are expressed in the capsule [29]. However, the exact role of these molecules for the maintenance of the adrenal cortex is currently unknown. It was originally suggested that GLI3 might have a role in adrenal development, as the expression of a truncated GLI3 with constitutive transcriptional repressor activity leads to the development of Pallister–Hall syndrome in human, which also included adrenal hypoplasia or aplasia in some cases [145]. Mouse bearing a similar *Gli3* allele was also first reported to have adrenal aplasia [87] but a subsequent study using the same model did not observe this phenotype [88].

**Figure 3:**
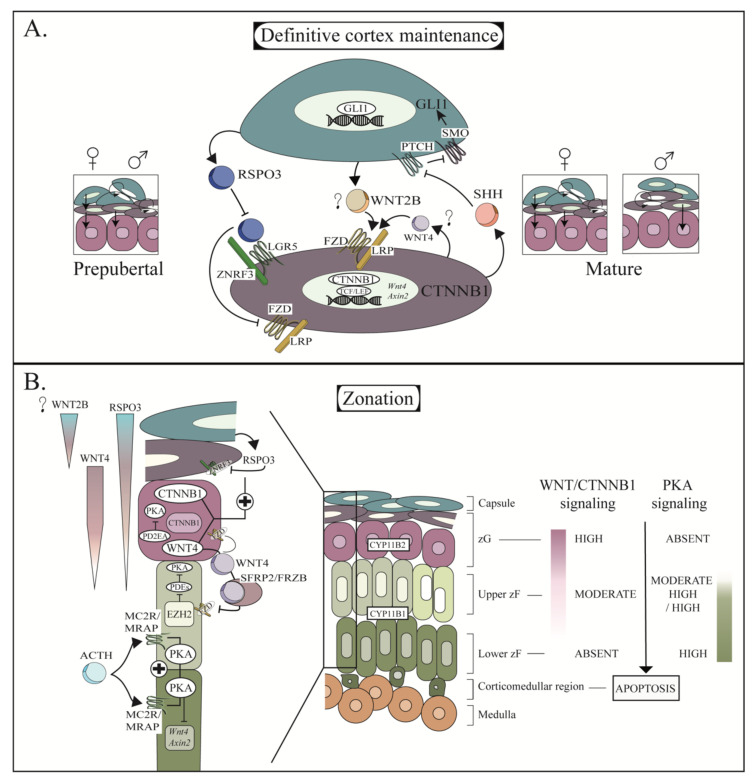
Development, maintenance, and establishment of zonation in the mice definitive adrenal cortex: (**A**) Schematics model for the definitive cortex maintenance. A WNT/SHH double-paracrine mechanism is needed to ensure proper development and maintenance of the adrenal cortex. Capsular stem cells regulate WNT pathway activation in the progenitor zG cells by secreting RSPO3, which in turn induces the clearance from the cell membrane of the WNT signaling inhibitor ZNRF3 and its subsequent degradation. This leads to an increase in canonical WNT signaling in the subcapsular progenitor and it promotes their proliferation (and subsequent differentiation into steroidogenic zG cells). Capsular WNT2B could be the main ligand responsible for the maintenance of the adrenal cortex (as WNT4 expressed in the zG appears to be more important for the differentiation of the steroidogenic zG cells). SHH secreted by the zG progenitor cells promotes the activation of GLI1 in the capsular stem cells and, consequently, their proliferation. Before puberty, both the capsular stem cells and subcapsular progenitor cells contribute to the maintenance of the adrenal cortex. After puberty, both the capsular stem cells and subcapsular progenitor cells contribute to the maintenance of the adrenal cortex in female. In male, only the subcapsular progenitor cells contribute to the maintenance of the adrenal cortex. (**B**) Schematics model for adrenal cortex zonation. Capsular and subcapsular WNTs and RSPO3 secretion create a gradient of WNT/CTNNB1 activity throughout the upper cortex. Elevated WNT/CTNNB1 activity drives steroidogenic zG cells differentiation and inhibits differentiation of zG cells into zF. PDE2A (whose transcription is positively regulated by CTNNB1) could be involved in the inhibition of PKA signaling in the zG by degrading cAMP. Moderate/low WNT/CTNNB1 favors the differentiation of zG cells into zF cells and the proliferation of zF cells. Binding of ACTH to its receptor promotes the activation of PKA signaling and antagonizes WNT signaling. Potential expression of FRZB or SFRP2 in the zF could also contribute to the inhibition of WNT signaling in the zF. PKA signaling is also optimized by adrenocortical cell programming by EZH2, which inhibits the transcription of *Pde7b, Pde3a* and *Pde1b*.

Although SHH acts on the *Gli+* capsular cells, it was also shown that the *Shh*^flox/flox^; *Nr5a1*-*Cre* animals had fewer proliferating cells in the outer layer of the adrenal cortex [29]. Moreover, tracing experiments demonstrated that the *Shh+* cells were also able to proliferate and move inward centripetally, indicating that they also are progenitor cells [28]. These results led to the establishment of the two progenitor lineages model in which Gli+ cells (later coined the adrenal stem cells) give rise to both steroidogenic cells and subcapsular progenitor cells, which secrete SHH to allow the proliferation/maintenance of the capsular stem cells. Shh+ cells also proliferate and further differentiate into steroidogenic cells upon their centripetal migration (Figure 3A) [28]. Subsequent experiments demonstrated that both the capsular stem cell and the subcapsular progenitor cell populations were also important to maintain homeostasis of the adrenal cortex in prepubertal animals (males and females) and mature females, while the subcapsular progenitor cells were the main contributors to adrenal homeostasis in mature males (Figure 3A) (though capsular stem cells maintain a role when important regeneration is needed) [53,54]. The importance of sexual dimorphism for the maintenance of the adrenal cortex will be discussed in a later section of this review.

Aside from the Hedgehog signaling, the canonical WNT signaling also has an important role for the development and the maintenance of the definitive cortex. This role was first suggested when a transgenic mouse model inactivating *Ctnnb1* (the main effector of canonical WNT signaling) in the adrenal cortex was generated. In this model, the adrenal gland initially formed. However, the adrenal cortex was unable to grow properly, leading to the complete atrophy of the adrenal gland by e18.5 [16]. Using a similar model in which *Ctnnb1* recombination was not as efficient, it was demonstrated that CTNNB1 was also essential for the maintenance of the adult adrenal cortex [16]. It was suggested at that time by the authors that the changes observed in the latter model could be associated with the depletion of a population of progenitor cells [16]. The role of CTNNB1 signaling in the progenitor cell population was later confirmed using lineage tracing experiments [53]. Indeed, it was demonstrated that subcapsular cells expressing *Axin2* (a transcriptional target of CTNNB1) contribute to adrenal cortex regeneration following dexamethasone-induced adrenocortical atrophy [53]. Furthermore, inactivation of Ctnnb1 in Axin2+ cells using the *Axin2^CreERt2^* mouse strain led to inefficient regeneration of the adrenal cortex, reduced *Shh* expression and reduced expression of *Ctnnb1* target genes, demonstrating that at least some progenitor cells were *Shh^+^, Ctnnb1^+^* [53].

If it was demonstrated that canonical WNT signaling induced SHH signaling in the subcapsular adrenal cortex (which led to the proliferation of the capsular *Gli1+* cells), it was also demonstrated that the effect was reciprocal and that *Gli1+* cells induced canonical WNT signaling in the *Shh+* cells (Figure 3A). Indeed, it was first shown that *Gli1+* cells express and secrete *Rspo3*, an enhancer of WNT signaling [6]. It was further demonstrated that the inactivation of *Rspo3* at different time points between e11.5 and 12-week-old animals (using either a ubiquitously expressed tamoxifen Cre inducible mouse strain or a *Gli1* regulated tamoxifen Cre inducible mouse strain) results in developmental and maintenance defects of the definitive cortex. These defects were associated with the loss of CTNNB1 target genes and *Shh* expression in the subcapsular cells as well as the loss of capsular *Gli1* expression [6].

It was also demonstrated in other systems that RSPOs promote the clearance of the inhibitor of WNT signaling ZNRF3 from the cell membrane [105,146]. Interestingly, inactivation of *Znrf3* in the developing adrenal cortex (using the *Nr5a1*-cre strain) or in the zG of adult animal (using the AS*^Cre^* strain) increases WNT/CTNNB1 signaling and promotes adrenal hyperplasia (Figure 3A) [23]. Together, these findings indicate that inactivation of ZNRF3 by RSPO3 is essential for the development and maintenance of the adrenal cortex. Regulation of the canonical WNT signaling by *Gli1+* cells was further confirmed as it was demonstrated that CTNNB1 transcriptional activity and adrenocortical regeneration were improved in a mouse model in which a constitutively activated form of SMO is expressed in capsular stem cells following dexamethasone-induced adrenocortical atrophy [53]. Previously mentioned experiments suggested that canonical WNT signaling play a key role in the maintenance of the adrenal cortex. However, 19 WNTs have been identified in the mice suggesting that numerous WNTs could be involved in this process. WNT4 is strongly expressed in the subcapsular region of the adrenal cortex as early as e14.5 [110]. Interestingly, inactivation of *Wnt4* either globally [110] or specifically in the adrenocortical cells [6,25] affects steroidogenic zG cells, CYP11B2 expression and aldosterone secretion [25,110], but only has a marginal effect on adrenal size and does not appear to have an important effect on the proliferation or maintenance of the adrenal cortex. This suggests that WNT4 is more important for the zonation of the adrenal cortex than for the proliferation of the progenitor cell population, and that other WNTs are more important for the proliferation of the progenitor cells (Figure 3A,B). This could also suggest that other WNTs compensate for the loss of WNT4 or act in synergy with WNT4 to regulate the proliferation of the progenitor cells. *Wnt2b* expression has been detected in the adrenal capsule as early as e13.5 [147] and could therefore be the main WNT ligand involved in the crosstalk between the capsular stem cells and the subcapsular progenitor cells. Taken together, all these studies demonstrate that a WNT/SHH double-paracrine mechanism is needed to ensure proper development and maintenance of the adrenal cortex via regulating the capsular stem cell population and the subcapsular progenitor cell population (Figure 3A).

Whether the Hedgehog and WNT signaling pathways appear to be the most important pathways involved in the development and maintenance of the definitive cortex, other biological processes and signaling pathways are also involved. FGFR2 and INSR/IGF1R, which have been shown to respectively have a role during development of the fetal cortex [10] and AGP formation [89], could also participate to the development of the definitive cortex. Inactivation of the *Fgfr2* isoform IIIb (which is expressed in the subcapsular region of the developing adrenal cortex) impairs the development of the adrenal cortex by potentially affecting cell proliferation [85]. On the other hand, inactivation of *Insr1* and *Igf1r* using the h*Cyp11a1*-*iCre* gives rise to small abnormal adrenal glands in adult animals that produce less corticosterone and that require exogenous sodium supplementation for their survival [39]. MicroRNAs also play an important role in the development of the definitive cortex, as the inactivation of Dicer using a *Nr5a1*-*Cre* strain leads to adrenal failure at birth associated with the progressive atrophy of the adrenal cortex starting at e16.5 caused by an increase in cellular apoptosis [17,18]. One common denominator with these transgenic mouse models is that development and maintenance defects are associated with a decrease in the expression of NR5A1, or in a decrease in the number of NR5A1+ cells. Although these latter observations could simply indicate that fewer steroidogenic cells are present in these animals, it is likely that the loss of NR5A1 in these models contributes to the observed phenotype. Inactivation of *Nr5a1* could not be achieved in the definitive cortex (contrary to what was observed in the fetal cortex) using a h*Cyp11a1*-*iCre* strain, suggesting that the lack of *Nr5a1* is incompatible with the definitive cortex development [40]. Furthermore, inactivation of *Nr5a1* using the *AS^cre^* strain also leads to the loss of the zG suggesting again that *Nr5a1* expression is essential for adrenocortical steroidogenic cell maintenance [48]. Finally, overexpression of rat *Nr5a1* in transgenic mouse models leads to nodule formation [99] while overexpression of *NR5A1* in the human H295R adrenal cell lines increases cellular proliferation [99]. Together, these studies suggest that NR5A1 maintains the definitive adrenal cortex by regulating the proliferation of the steroidogenic cells. Interestingly, loss of NR5A1 SUMOylation also leads to ectopic expression of *Shh* in the developing testes, increased *Shh* expression in the adrenal cortex and the presence of *Shh*+ cells deep into the adrenal cortex [98]. Experiments performed in the embryonic cell line mHypoE-40 further demonstrated that SUMOylation modulated the DNA binding of NR5A1 to the promoter of *Shh*. This suggests that NR5A1 activity must be tightly regulated by SUMOylation to properly regulate the expression of *Shh* in adrenocortical cell populations.

Finally, postnatal impairment of adrenocortical maintenance was also observed following the inactivation of the effectors of the Hippo signaling, *Yap* and *Taz,* in steroidogenic cells [32], or global inactivation of *Nr0b1* [93]. In these two models, degeneration of the adrenal gland was also associated with the appearance of large multinucleated lipoid structure in the adrenal cortex potentially caused by a decrease in the progenitor reserve or progenitor cell reserve [32,93], as suggested by a decrease in the expression of *Shh* following the inactivation of *Yap*/*Taz* [32]. A potential link between these two models is also suggested by the fact that inactivation of *Yap/Taz* also leads to a decrease in the expression of *Nr0b1* [32]. Interestingly, the degeneration of the adrenal cortex was only observed in male following the inactivation of *Yap/Taz* [32] suggesting that maintenance of the adrenal cortex is sexually dimorphic. Surprisingly, inactivation of the main kinases of Hippo signaling, *Lats1* and *Lats2* (which lead to an increase in YAP and TAZ activity), does not lead to hyperplastic adrenal gland or increased proliferation of the progenitor cells, but rather leads to the transdifferentiation of adrenocortical cells into myofibroblast-like cells [33].

## 8. Establishment of Zonation: The Opposing Roles of WNT and PKA Signaling Cascades

One key feature of the adrenal cortex is the appearance of concentric zones in which aldosterone (zG) and corticosterone (zF) are synthetized in mice, and aldosterone (zG), cortisol (zF) and DHEA/DHEAS (zR) are synthetized in humans. In mice, functional zonation (as shown by the activity of the *Cyp11b2* promoter) is first observed at e16.5 in rare scattered subcapsular cells (with a similar pattern being also observed at 1dpp) [48]. However, this limited number of cells might reflect a delay in the appearance of the fluorescent reporter marker following recombination. Even though the presence of the enzyme necessary for mineralocorticoid production only appears in late stages of adrenal cortex development, zonation of other genes such as *Wnt4* precedes this time point and can be observed as early as e14.5 in the outer cortex [110]. This suggests that WNT signaling is not only important for adrenal cortex development/maintenance, but also for proper zonation. The importance of WNT signaling for zG development was first suggested when it was demonstrated that *Wnt4* global knockout animals secreted less aldosterone [110]. This result was later confirmed by the inactivation of *Wnt4* using a *Nr5a1-cre* strain in which the expression of zG markers was reduced [6,25], and an expansion of the zF was observed [25]. The loss of *Wnt4* also led to a reduction in the expression of CTNNB1 [25] and its downstream target *Axin2* [6] suggesting that canonical WNT/CTNNB1 signaling is involved in the differentiation of the zG (Figure 3B).

The importance of CTNNB1 in this process was confirmed by the study of transgenic mouse models in which expression of CTNNB1 was stabilized. If the stabilization of CTNNB1 at an early time point of adrenal development (either by inactivating *Apc* or by expressing a constitutively active form of CTNNB1 that lack the phosphorylation sites on exon 3 necessary for its degradation) led to significant adrenal hypoplasia during development [15,17], its stabilization at later time points (either by inactivating *Apc* with a less efficient *Nr5a1-cre* [15] or by expressing the *Ctnnb1^ex3^* allele in *Akr1b7*+ cells [46]) rather led to an increase in cellular proliferation and adrenal dysplasia. Even more important for the zonation process, CTNNB1 stabilization in adrenocortical cells led to the downregulation of the zF marker AKR1B7, expression of ectopic CYP11B2+ cells in the zF and hyperaldosteronism [46]. The importance of CTNNB1 was further confirmed in the H295R cell line as its inactivation also decreased aldosterone production in these cells [107]. Recent studies also demonstrate that ectopic CTNNB1 activation in zG cells blocks their differentiation into zF cells, increases aldosterone production and the number of rosettes (structures adopted by glomerular cells) [49,50]. Furthermore, *Ctnnb1* inactivation in zG cells reduced rosette frequency, though this might be associated with the role of CTNNB1 at cellular junctions rather than in WNT signaling [49] as membranous CTNNB1 is also lost following inactivation of *Fgfr2*, which also leads to the impairment of rosette formation [49]. Finally, global inactivation of the WNT signaling inhibitor *Sfrp2* leads to the appearance of CTNNB1/CYP11B2 positive cells in the zF [107], while the inactivation of the previously mentioned capsular activator of WNT signaling *Rspo3* leads to the loss of all zG markers [6]. Together, these findings clearly indicate that WNT signaling is the key pathway regulating zG cell differentiation (Figure 3B).

If WNT signaling plays a key role in the zG formation and aldosterone production, ACTH/protein kinase A (PKA) signaling is the most important pathway for corticosterone synthesis by the zF. The role for this pathway for the differentiation of the zG cells into zF cells was also recently demonstrated. First, it was demonstrated that zF expansion and expression of zF markers are induced by ACTH treatment in mice, while *Cyp11b2* expression and the activity of a WNT signaling reporter transgene is extinguished [25]. In addition, forskolin (a known pharmacological activator of adenylate cyclase leading to PKA signaling activation) decreases *Wnt4* and *Axin2* expression in H295R cells [25]. This role was further confirmed in a transgenic mouse model inactivating *Prkar1a*, the gene encoding the regulatory subunit type 1a of PKA, which leads to the constitutive activation of PKA in the adrenal cortex. In this model, PKA activation leads to the expansion of zF and an important repression of CTNNB1 activity and *Cyp11b2* expression [25]. Consistent with the role of ACTH for zF maintenance, inactivation of *Mrap* (an accessory protein essential for MC2R, the ACTH receptor) activity leads to neonatal lethality due to the absence of corticosterone secretion [91]. However, surviving animals (following corticosterone replacement treatments) presented hypoplastic adrenals that did not express CYP11B1 [91]. Furthermore, WNT4 and CTNNB1 expression was detected in most cells of the remaining cortex though CYP11B2 was only expressed in a portion of these cells [91]. Similarly, to the inactivation of *Mrap*, the inactivation of *Mc2r* also leads to neonatal lethality in most animals, with rare animals surviving through adulthood [90]. The few surviving animals had hypoplastic adrenal with CYP11B2 expressing zG cells still present (despite lower circulating aldosterone levels) (Figure 3B) [90]. WNT signaling was however not evaluated in this model. Finally, it was demonstrated that global SUMOylation was negatively regulated by PKA signaling and positively by CTNNB1 in the adrenal cortex. However, a possible role for SUMOylation in the zonation process has not yet been identified [24].

Epigenetic factors can also contribute to zF differentiation, as it was demonstrated that the inactivation of the histone methyltransferase *Ezh2* affects zF differentiation leading to an expansion of the zG [19]. The effect of the loss of *Ezh2* on zF differentiation was further associated with a loss of PKA activity that was associated with an increase in the expression of negative regulators of PKA including the *Pde7b, Pde3a* and *Pde1b* [19] (Figure 3B). Interestingly, CTNNB1 was also shown to positively regulate the expression of the phosphodiesterase *Pde2a* in the zG, suggesting that phosphodiesterases contribute to the inhibition of PKA signaling in the zG [50]. The contribution of other PDEs for cAMP/PKA signaling inhibition in the adrenal cortex was also demonstrated using both mouse models and adrenal cell lines. Indeed, it was demonstrated that *Pde8b^−/−^* have elevated levels of serum and urinary corticosterone [102,103], and that inactivation of *Pde8b* potentiates steroidogenesis and corticosterone steroidogenesis in Y1 cells and H295R cells by potentially increasing cAMP levels [102,103]. The cAMP levels were also higher in the adrenal gland of hypomorphic *Pde11a^−/−^* mice. Furthermore, *Pde11a^−/−^* mice failed to suppress corticosterone in response to low dose dexamethasone [104].

Whether most studies indicate that WNT signaling is essential for zG differentiation and that PKA signaling is essential for zF differentiation, one study demonstrates that WNT signaling also contributes to zF maintenance. Indeed, inactivation of the WNT signaling inhibitor *Znrf3* promotes the hyperplastic growth of the zF rather than the expected zG growth [23]. It was further confirmed that this hyperplasia is caused by an increase in WNT/CTNNB1 signaling as inactivation of *Porcn* (an O-acyltransferase required for post-translational modification of all WNTs necessary for WNT secretion and activity) concomitantly with *Znrf3,* rescuing the phenotype observed following *Znrf3* inactivation. The Concomitant inactivation of one copy of *Ctnnb1* with *Znrf3* also leads to reduced proliferation and adrenal cortex size following *Znrf3* inactivation [23]. Revisiting the expression of *Wnt4* and CTNNB1, it was demonstrated that a gradient of expression is normally observed in the adrenal cortex going from high expression in the zG to moderate expression in the upper (or outer) zF to no expression in the lower (or inner) zF [23]. Following the deletion of *Znrf3*, moderate *Wnt4* and CTNNB1 expression could be seen throughout the cortex suggesting that not only WNT4/CTNNB1 signaling is responsible for the phenotype observed following the inactivation of *Znrf3*, but that it is also normally involved in the proliferation of upper zF cells [23] (Figure 3B).

The gradient of expression of WNT4/CTNNB1 signaling in the zF from mild expression in the upper zF to its extinction in the lower zF also illustrates the fact that the zF is heterogenous. The presence of different zones (based on gene expression) in the zF was also observed in other studies. For example, by using single-cell transcriptomics analysis (and confirmed by RNAscope analyses) it was demonstrated that a specific cell population formed the lower zF [148]. Furthermore, this cell population, named zFasc1 by the authors, significantly expands in response to chronic stress exposure [148]. Interestingly, it was demonstrated that *Abcb1b*, one of the genes overexpressed in the zFasc1, positively regulates cortisol secretion [148] suggesting that zFasc1 cells could be more potent than cells from the upper zF. Another study that demonstrated that the lower zF differs from the upper zF (based on immunofluorescence for CYP2F2 and DHCR24 and RNAseq data) also suggested that the zF could potentially be divided into even more concentric zones, and that the lower zF shares some similarities with the X-zone [149]. Finally, it was also shown in that study that the lower zF expands in response to T3 treatment in females [149]. Interestingly, *Wnt4* expression decreased in the adrenal cortex of T3 treated mice while expression of *Mrap* was induced [149]. This last result again suggests that the WNT signaling gradient (and potentially PKA signaling) could be key for the establishment of these zF zones and their response to different challenges. Whether the upper and lower zF have partially different functions has, however, not yet been determined.

As mentioned previously, murine adrenal cortex only comprised two zones, the zG and the zF, and lacked the zR that synthetizes androgens in humans. For this reason, very few studies have evaluated the differentiation of zR cells. However, one study demonstrated that PKA signaling activation was involved in its formation. Indeed, inactivation of *Prkar1a* not only affects zG/zF differentiation, but also leads to the formation of a third zone next to the medulla [26,37]. While a study using the *Ark1b7*-*Cre* model to inactivate *Prkar1a* initially suggested that this zone could correspond to the resurgence of the X-zone [26], a subsequent study combining the inactivation of *Prkar1a* using the *AS*^cre^ strain and tracing experiments demonstrated that this third zone does not correspond to the resurgence of the X-zone, but rather arises from the differentiation of the lower zF [37]. In this model, the authors demonstrated that expression of CYP17 and its regulator CYB5 could be detected in this zone, and that adrenal cortex from mutant animals could synthetize DHEA/DHEAS [37]. These results indicate that this zone resembles human zR [37]. Interestingly, a persistence or resurgence of the X-zone was also observed in *Pde11a^−/−^* mice [104]. As the inactivation of both *Pkrkar1a* and *Pde11a* increase cAMP/PKA signaling, there is a possibility that the observed X-zone in the in *Pde11a^−/−^* mice could also resemble the human zR. It would therefore be interesting to determine if the adrenal of *Pde11a^−/−^* mice can produce DHEA/DHEAS.

Another study demonstrated that overexpression of human DENND1A.V2 (a truncated isoform of a clathrin-binding protein that has not been detected in rodents but is expressed in human H295A cell line [150]) in a transgenic mouse model leads to an important increase in the expression levels of adrenal *Cyp17a1*, despite only low levels of *DENND1A.V2* being detected [84]. However, it was not determined if a zR was formed in these mice. Nonetheless, these findings correlate with the role of DENND1A.V2 in the hyperandrogenemia associated with polycystic ovarian syndrome (PCOS) in women [151,152,153]. The exact mechanism of action of DENND1A.V2 is still unknown, but two mechanisms were proposed for its role in PCOS theca cells. First, it was suggested that nuclear DENND1A.V2 could activate the transcriptional activity of *Cyp17a1* either by facilitating the transport of ligand/receptor to the nucleus or by acting as a scaffold for transcription factors [151,152,153]. Another possibility is that DENND1A.V2, which has a clathrin-binding domain, could regulate (either directly or by interfering with the action of DENND1A.V1) the internalization/endocytosis/recycling of GPCR and therefore increase cAMP/PKA signaling [151,152,153]. This could lead to the formation of a zR in the mice as observed following *Prkar1a* inactivation [37]. However, again, cAMP/PKA activity was not evaluated in mice overexpressing *DENND1A.V2* [84].

Finally, although it is usually thought that murine adrenal cortex does not produce androgen, it has been demonstrated that the spiny mouse expresses *Cyp17a1* and produces DHEA [154]. More recently, it was also shown that the adrenal gland of C.B.-17 SCID mice also produces a low level of DHEA and its downstream metabolite, suggesting that some strains of mice can actually produce androgens [155]. Again, the presence of a zR in C.B.-17 SCID mouse was not evaluated. Nonetheless, this suggests that some mouse strains could be useful to study zR differentiation and functions.

Tracing experiments (13,27,28,36,37,48,53) have demonstrated that maintenance and zonation are usually linked in the adrenal cortex in a process in which capsular stem cells and subcapsular progenitor cells move inward centripetally and differentiate into zG cells. The latter will then differentiate into zF cells before dying by apoptosis at the junction between the adrenal cortex and the medulla. If this is normally the case, tracing experiments have demonstrated that maintenance of the adrenal cortex and zonation can be separated from one another in certain contexts. For example, inactivation of *Nr5a1* in *Cyp11b2+* zG cells leads to the loss of the zG without affecting zF maintenance [48]. Furthermore, zF cells were no longer derived from zG cells in this model [48]. Overexpression of CTNNB1 in the *Cyp11b2+* zG cells also blocks the differentiation of zG cells into zF cells, leading to the maintenance of a zF no longer derived from the zG [50]. Numerous hypotheses could explain the separation of zG and zF maintenance. First, it is possible that the stem/progenitor cells can bypass their need to differentiate into zG cells before differentiating into zF cells, as recombination does not occur in stem/progenitor cells in these models. On the other hand, it is possible that the differentiation of zG to zF cells is accelerated in some models, and that recombination simply does not have time to occur in zG cells before their differentiation into zF cells. Another possibility is that proliferation of zF cells increases in these models. Residual WNT signaling could be involved in the proliferation of zF cells as observed following the inactivation of *Znrf3* [23]. A fourth possibility is that other populations of normally inactive stem/progenitor cells are present in the adrenal cortex and take over in this context. For example, it was shown in rat that cell expressing POU5F1, a marker of stem cells, could be seen throughout the adrenal cortex before puberty with the number of POU5F1+ cells increasing in the zG after puberty and decreasing in the rest of the cortex [156]. The presence of POU5F1+ cells has not been evaluated in the mouse, but the presence/maintenance/replication of these prepubertal POU5F1+ cells could be maintained in the adult zF in abnormal conditions. Finally, it was demonstrated that a population of Nestin+ cells, mainly located in the subcapsular region (with rare cells also observed in the zF) also has characteristics of stem/progenitor cells and can differentiate into steroidogenic cells of the zG and the zF [60]. These cells lacked co-staining with GLI1 and SHH, suggesting that they are a different population of stem/progenitor cells, though they could potentially still be descendants of GLI1 or SHH positive cells. More interestingly, these cells do not seem to play an important role in the normal maintenance of the adrenal cortex, but their differentiation into steroidogenic cells increases following stress [60], again suggesting that “dormant” progenitor cells could be present in the adrenal cortex and ready to respond if necessary.

## 9. Sexual Hormones Play an Important Role in the Maintenance of the Adrenal Cortex

**Figure 4:**
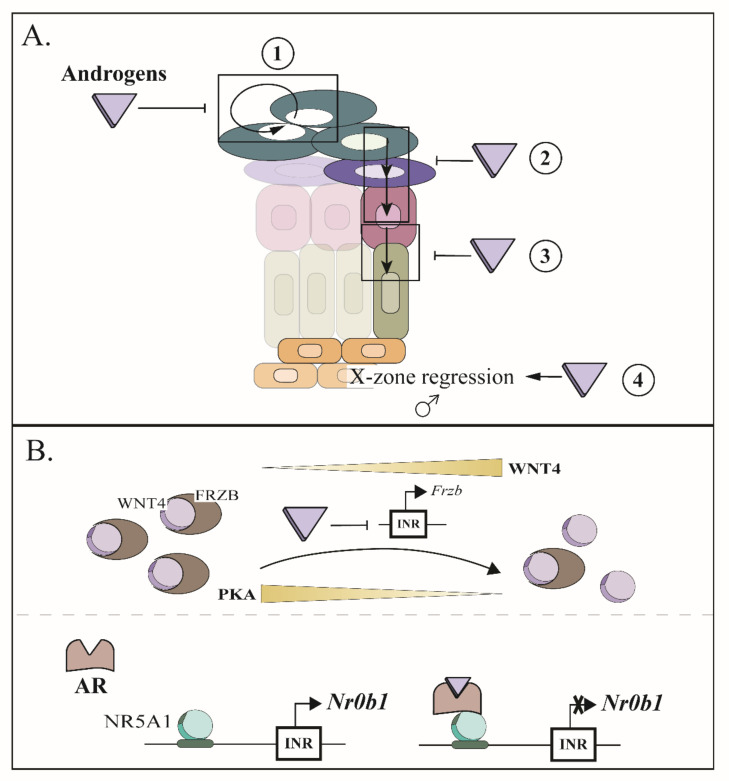
Androgens affect adrenocortical maintenance: (**A**) Androgens act at several levels to regulate the maintenance of the adrenal cortex. ① Androgens can limit cortical cell turnover by reducing the proliferation of the capsular stem cells; ② abolish the contribution of the capsular stem cells to the steroidogenic lineage; ③ limit the differentiation of zG cells into zF cells; and ④ permit the complete regression of the X-zone in male mice. (**B**) Potential molecular action of androgen or its receptor in the adrenal cortex. Global molecular mechanisms regulated by androgens are unclear. It was suggested that androgens could stimulate WNT signaling (and potentially restrain PKA signaling) via the downregulation of the WNT inhibitor *Frzb*. Liganded AR can also negatively regulate the transcriptional activity of *Nr0b1* by binding with NR5A1.

As previously stated, the adrenal gland of mouse is sexually dimorphic. Indeed, the adrenal glands of female have a higher weight than their male counterparts [134,157], which could be explained, in part, by the regression of the X-zone at puberty in male and its maintenance in female until the first gestation [134,135], and in part by the fact that the volume of the zF (but not the zG) is higher in females [134]. Furthermore, the higher volume of the zF was also associated with higher levels of circulating corticosterone [134]. Interestingly, it was shown that castration of mature male mice leads to the appearance of a secondary X-zone [158], while testosterone treatment caused the rapid disappearance of the X-zone in females [159]. These findings demonstrate the key role played by male hormones in the adrenal cortex (Figure 4A).

The importance of sexual dimorphism and the roles of androgens for the maintenance of the adrenal cortex were also demonstrated more recently (Figure 4A). By using lineage-tracing experiments of the zG *Axin2*+ or *Wnt4+* cells, it was first shown that complete renewal of the adrenal cortex was faster in females (approximatively 3 months) than in males (estimated at 9 months) [54]. Furthermore, lineage-tracing experiment of the capsular *Gli1+* stem cells revealed that these cells contribute to the steroidogenic lineage in both sexes in 3-week-old animals, but that after puberty the contribution of the capsular stem cells to the steroidogenic lineage was almost completely abolished in males but that this contribution remains important in females [54] (Figure 3A, Figure 4A). Again, it was demonstrated that androgens play a key role in this process as recruitment of *Gli1+* was accelerated in orchiectomized males, while treatment of ovariectomized females with dihydrotestosterone (DHT) had the opposite effect [54]. Adrenal cell renewal was not affected following ovariectomy, suggesting that progesterone and estrogen do not contribute to this process [54]. Interestingly, contribution of *Gli1+* cells to adrenal cell renewal in male could be observed in a model of adrenal regeneration (following dexamethasone treatment), suggesting that these cells can serve as a reservoir in male when important adrenal recovery is needed [53].

The molecular mechanisms of androgen action in adrenal maintenance are not well defined. However, it was demonstrated that gonadectomy decreases the expression of CTNNB1 target genes and dramatically increases the expression of the inhibitor of WNT signaling *Frzb*, while DHT supplementation has the reverse effect [37] suggesting that androgens block the differentiation of zG cells into zF cells or limit the proliferation of the upper zF cells (Figure 4A, B). Another potential target of androgen is *Nr0b1* whose expression is higher in the adrenal cortex of females (compared to males), and whose expression increases in orchiectomized males [160]. Interestingly, it was demonstrated that liganded AR negatively regulates the transcriptional activity of *Nr0b1* by binding with NR5A1 [160] (Figure 4B). YAP have also been shown to bind to AR in prostate cancer [161], suggesting that Hippo signaling could be involved in the regulation of androgen activity in the adrenal cortex. Finally, effects of the loss of *Ar* in the adrenal cortex were evaluated recently. As expected, an abnormal retention of the X-zone could be observed in males [44]. A tendency for higher circulating corticosterone levels in aging animals is also observed in these animals. Interestingly, divergent results could be seen in mutant animals and their orchiectomized counterparts. For example, a reduction in the zF marker ARK1B7 and an increase in apoptosis of zF cells were solely observed in orchiectomized/AR negative animals suggesting that androgens do not exclusively act via AR [44]. Contrary to what is observed in males, ARK1B7 expression was lost in the adrenal cortex of female mutant animals [43] and X-zone regression was independent of AR [43]. Further delineation of the molecular mechanisms of action of AR is still needed to comprehend its function in the adrenal cortex.

Recently, a potential new role for androgen action on adrenal cortex homeostasis has emerged. Indeed, using the *Znrf3 ^flox/flox^; Nr5a1-Cre*^high^ mouse model, it was first demonstrated that 78-week-old females were developing metastatic adrenocortical carcinoma (ACC) while no such tumors were observed in males [111]. It was further demonstrated that tumor development in males was blunted by the induction of adrenocortical cells senescence, followed by the recruitment and differentiation of phagocytic macrophages [111]. Hyperplasia regression in male was confirmed to be androgen dependent, as the male phenotype (early recruitment of phagocytic macrophages and regression of initial hyperplasia) was recapitulated in females implanted with testosterone pellets [111]. These results might in part explain why ACC incidence is higher in women than in men [162,163,164]. Interestingly, macrophage number also increases following chronic stress exposure [148]. It was also suggested that adrenal macrophages control lipid metabolism in both sexes, and that macrophage depletion in the whole animals (performed in females) leads to lower local production of aldosterone in stressed animals [165]. Furthermore, sexual dimorphism of the adrenocortical macrophage populations is also observed in wild-type mice as MCH class II^low^ macrophages are solely present in females and are dependent of the X-zone [165]. Together, these studies suggest that macrophages are important to maintain adrenal homeostasis. Further studies are warranted to thoroughly understand their mechanisms of action.

Function of estrogen in adrenal cortex maintenance has not been studied as much as androgens. However, one study using the estrogen-deficient aromatase knockout mouse models suggests that estrogen deficiency leads to the inhibition of telomerase activity, telomere shortening of cortical cells, and a decrease in cell proliferation in the female adrenal cortex [69], while another study also suggests that estrogen might influence *Nr0b1* regulation [160]. Again, further studies will be needed to evaluate the role of female hormones in adrenal cortex maintenance.

Finally, a sexually dimorphic response to the thyroid hormones T3 is observed in the adrenal cortex of prepubertal animals [149]. While differences in this response could be partially explained by the initiation of the X-zone regression in males, the expansion of the inner zF in females suggests that T3 also contributes to the dimorphism of the definitive adrenal cortex. Estrogen and T3 action demonstrate that although androgen action at puberty is probably the most important factor regulating adrenal gland sexual dimorphism, other factors also contribute to this dimorphism.

## 10. Emergence of Spindle-Shaped Cells in Aging Mice

One last feature commonly observed in older mice is the accumulation of subcapsular non-steroidogenic spindle-shaped cells, named A cells. Further differentiation of a few of these cells in large lipid-laden sex-steroid producing cells, named B cells, is also observed in some mouse strains [166,167,168]; this is a process that is greatly enhanced following gonadectomy [55,169]. Accumulation of these cell types is often considered a gonad-like tumor as these cells express gonad markers such as *Cyp17a1*, *Gli1, Lhcgr* and *Amhr2* [13,55]. Premature appearance of the spindle-shaped cells is also observed following the inactivation or overexpression of numerous genes in the adrenal cortex [13,19,26,31,34,43,44,46,47,71,99,103,104]. Because it was demonstrated that the spindle-shaped cells also express *Gata4* [19,31,46,170] and *Wt1* [13,19], it has been suggested that these cells constitute a population of progenitor cells sharing similarities with AGP cells, which accumulate as an attempt to maintain homeostasis in animals with adrenal insufficiency. Further differentiation of A cells to B cells has also been associated with elevated LH secretion [70,170,171,172]. Evaluation of LH levels (and other sex hormones) in the different mouse models presenting this phenotype is therefore essential to determine if the appearance of these cells is intrinsic to the adrenal or secondary to the inactivation of these genes in other steroidogenic tissues such as the ovary, testis, or pituitary.

Currently, two theories, both based on tracing experiments, have been suggested for the origin of the A cells. The first theory suggests that both capsular *Wt1+* [13] and *Gli1+* [55] cells can form A cells following gonadectomy. Blocking GLI1/2 activity with a pharmacological inhibitor following gonadectomy also decreases the expression of gonad markers, further confirming the results obtained with the tracing experiments [55]. More recently, the origin of these cells was also evaluated following the inactivation of *Ezh2* [19]. In this model, tracing experiments rather suggested that *Nr5a1*+ cells dedifferentiate into A cells [19]. Furthermore, in this model, expression of GATA4 preceded the expression of WT1 in the adrenal cortex, suggesting that GATA4+ cells do not originate from cells expressing WT1 in this model [19]. GATA4 expression was also broader than WT1 expression suggesting that GATA4 induction is independent of WT1 induction [19]. The different origin of the spindle-shaped cells could be associated with differences between the models (gonadectomy vs. aging mice) but capsular *Gli+* cells have also been shown to contribute to A cell formation in aging animals [55]. It is therefore possible that both the differentiation of capsular stem cells and dedifferentiation of *Nr5a1*+ cells contribute to A cell formation. Which *Nr5a1*+ cells (*Nr5a1*+, *Shh*+ subcapsular progenitor cells or *Nr5a1*+ steroidogenic cells) contribute to A cell formation and what would be the exact contribution of capsular cells and adrenocortical *Nr5a1*+ cells to A cell formation also remain to be determined.

No matter the origin of the spindle-shaped cells, GATA4 appears to play a prominent role in their appearance and their differentiation into B cells. Indeed, it was first demonstrated that the appearance of A cells is delayed and that their differentiation into B cells is blocked in ovariectomized *Gata*^+/−^ mice [86]. Conditional deletion of *Gata4* in the nascent forming A cells of ovariectomized mice also limits their proliferation and blocks their differentiation into B cells [86]. Furthermore, GATA4 appears to be the main factor responsible for the appearance of gonad-like tumor observed in *Inha*^−/−^ animals [172]. While inactivation of *Gata4* has been shown to block the formation of these cells, ectopic expression of GATA4 in the adrenal cortex (under the control of the *Cyp21a1* promoter) has also been shown to lead to the appearance of A cells in females and accelerate the appearance of both A and B cells following gonadectomy in males and females [71].

## 11. Conclusions

In the last decades, transgenic mouse models have been the driving force behind our understanding of the molecular mechanisms regulating the development, zonation and maintenance of the adrenal cortex. Combination of transgenic mouse models with genome-wide profiling of transcriptomes and epigenomes at the cellular levels further offers the possibility to comprehend the interplay between gene expression, transcription factors and chromatin state to uncover the gene networks regulating adrenocortical cell fate commitment. Disruption of the development, zonation and maintenance of the adrenal cortex have been associated with diseases such as Cushing syndrome, Carney complex and both adrenocortical adenoma and carcinoma. To fully understand these mechanisms could therefore lead to new therapeutic strategies to treat these pathologies.

## Figures and Tables

**Table 1:** Cre mouse strains used to study adrenal cortex development and maintenance.

Mouse Strains	Ref (Mouse Dev.)	Targeted Tissues/Cells during Adrenal Development and Maintenance	Targeted Genes (Inactivated)/Tracing Cell Populations	Ref
*CAG-CreER*	[4]	Global inactivation	*Gata4*	[5]
			*Rspo3*	[6]
*Osr1^eGFP-CreERt2^*	[7]	Coelomic epithelium	*Gata4*	[5]
			Tracing intermediate mesoderm descendants	[8]
*Tbx18^Cre^*	[9]	Coelomic epithelium	*Fgfr2*	[10]
*Gata4^CreERt2^*	[11]	Coelomic epithelium/AGP		
*Wt1^CreERt2/+^*	[12]	Coelomic epithelium/AGP Subpopulation of capsular cells	*Gata4*	[5]
			Tracing (capsular population)	[13]
			Tracing intermediate mesoderm descendants	[8]
*Nr5a1*-Cre^high^ (High transgene copy numbers)	[14]	AGP/fetal cortex, definitive cortex	*Apc*	[15]
			*Ctnnb1*	[16,17]
			*Ctnnb1ex3*	[17]
			*Dicer*	[17,18]
			*Ezh2*	[19]
			*Fgfr2*	[20]
			*Gata4*	[21,22]
			*Gata6*	[21,22]
			*Porcn*	[23]
			*Prkar1a*	[24,25,26]
			*Rnf43*	[23]
			*Shh*	[17,27,28,29]
			*Smo*	[28]
			*Wnt4*	[6,25]
			*Wt1 (activation)*	[13]
			*Znrf3*	[23]
*Nr5a1*-Cre^low^ (low transgene copy numbers)	[14]	AGP/fetal cortex, definitive cortex, few cells affected	*Apc*	[15]
			*Ctnnb1*	[15,16]
			*H19*	[15]
*Nr5a1*-Cre	[30]	AGP/fetal cortex, definitive cortex	*Gata6*	[31]
			*Yap/Taz*	[32]
			*Lats1/Lats2*	[33]
			*Mst1/Mst2*	[34]
*FAdE/Nr5a1-Cre*	[35]	Fetal cortex	Tracing fetal adrenocortical cells descendants	[35,36]
*FAdE/Nr5a1-CreERT2*	[35]	Fetal cortex	*Prkar1a*	[37]
			Tracing fetal adrenocortical cells descendants	[35,37]
*Nr5a1 ^eGFP-CreERt2^*	-	AGP/fetal cortex, definitive cortex		
*hCyp11a1-iCre*	[38]	Fetal cortex/definitive cortex	*Insr/Igf1r*	[39]
m*Cyp11a1-iCre*	[40]	Fetal cortex/definitive cortex	*Ctnnb1^ex3^*	[41]
			*Nr5a1*	[40]
*Cyp11a1^Gfp,Cre/+^*	[42]	Fetal cortex/definitive cortex	*AR*	[43,44]
*Akr1b7-Cre*	[45]	Fetal cortex/definitive cortex	*Ctnnb1^ex3^*	[24,25,46,47]
			*Prkar1a*	[25,26]
			*Prkaca*	[25]
*Cyp11b2^Cre^/*AS*^Cre^*	[48]	Aldosterone producing zG cells and their zF descendants	*Ctnnb1*	[49]
			*Ctnnb1^ex3^*	[49,50]
			*Ffg2r*	[49]
			*Prkar1a*	[37]
			*Nr5a1*	[48]
			*Nr0b1*	[48]
			*Znrf3*	[23]
			Tracing zG cell descendants	[23,37,48,50]
*Cyp11b1^eGFP-Cre^*	[51]	zF cells	*Cth*	[51]
*Gli1^CreERt2^*	[52]	Capsular stem cells	*Rspo3*	[6]
			*Smo (activation)*	[53]
			*Tracing capsular stem cell descendants*	[27,28,36,53,54,55]
*Shh^Cre^*	[56]	Subcapsular progenitor zG cells	Tracing subcapsular progenitor cell descendants	[28]
*Shh^CreERt2^*	[56]	Subcapsular progenitor zG cells	Tracing subcapsular progenitor cell descendants	[28,53]
*Axin2^CreERt2^*	[57]	WNT signaling activated zG cells	*Ctnnb1*	[53]
			Tracing zG cells descendants (including subcapsular progenitor cells)	[53,54]
*Wnt4 ^CreERt2^*	[58]	WNT signaling activated zG cells	Tracing zG cells descendants (including subcapsular progenitor cells)	[54]
*Nes-*CreERt2	[59]	Stress induced adrenocortical progenitor cells	Tracing stress induced progenitor cell descendants	[60]

**Table 2:** List of mouse models evaluating the development, zonation and maintenance of the adrenal cortex.

Genes	Mouse Models	Phenotype	Ref
*Apc*	*Apc ^flox/flox^; Nr5a1-Cre* ^high^	Adrenal hypoplasia	[15]
	*Apc ^flox/flox^; Nr5a1-Cre^low^*	Adrenal Hyperplasia, rare adenoma in older animals	[15]
	*Apc ^flox/flox^; Ctnnb1 ^flox/flox^; Nr5a1-Cre^low^*	Rescue of the hyperplasia	[15]
	*Apc ^flox/flox^; H19 ^floxDMD/floxDMD^; Nr5a1-Cre^low^*	Adrenal Hyperplasia with higher incidence of adenoma	[15]
*AR*	*Ar ^flox/Y^; Cyp11a1 ^Gfp,Cre/+^*	Abnormal retention of the X-zone, Subcapsular spindle-shaped cell hyperplasia	[44]
	*Ar ^flox/flox^; Cyp11a1 ^Gfp,Cre/+^*	Reduced expression of the zF markers AKR1B7, Subcapsular spindle-shaped cell hyperplasia	[43]
*Cbx2*	*Cbx2^−/−^*	Mild hypoplastic adrenal gland at e18.5	[81]
*Cited2*	*Cited2^−/−^*	Adrenal agenesis	[82,83]
	*Cited2^+/−^*; *Wt1^+/−^*	Adrenal and gonadal hypoplasia	[83]
*Ctnnb1*	*Ctnnb1 ^flox/flox^; Nr5a1-Cre* ^high^	Adrenal aplasia	[16,17]
	*Ctnnb1 ^flox/flox^; Nr5a1-Cre^low^*	Age-dependent adrenal cortex degeneration	[16]
	*Ctnnb1 ^flox/flox^; Axin2^CreERt2/+^*	Inefficient regeneration of the adrenal cortex	[53]
	*Ctnnb1 ^flox/flox^; AS ^Cre/+^*	Impaired rosette formation in the zG	[49]
	*Ctnnb1^ex3/+^; Nr5a1-Cre* ^high^	Adrenal agenesis (right adrenal), adrenal hypoplasia (left adrenal)	[17,25]
	*Ctnnb1^ex3/+^; Prkar1a ^flox/flox^; Nr5a1-Cre* ^high^	Partial rescue of the adrenal hypoplasia.	[25]
	*Ctnnb1^ex3/+^; Akr1b7-Cre*	Ectopic expression of zG cells at the expense of zF cells, hyperaldosteronism, Subcapsular spindle-shaped cell hyperplasia, rare adenoma in older animals	[46]
		Increased SUMOylation in the zF	[24]
	*Ctnnb1^ex3/+^; Prkar1a ^flox/flox^; Akr1b7-Cre*	Decreased WNT induced hyperproliferation and ectopic zG differentiation	[25]
	*Ctnnb1^ex3/+^; Prkaca ^+/−^; Akr1b7-Cre*	Accelerated WNT induced tumorigenesis	[25]
	*Ctnnb1^ex3/+^; Akr1b7-Cre, Akr1b7-Igf2*	Same phenotype as the *Ctnnb1^ex3/+^; Akr1b7-Cre* mice	[47]
	*Ctnnb1^ex3/+^; mCyp11a1-iCre*	Adenoma (Dab2+)	[41]
	*Nr5a1-Hoxb9; Ctnnb1^ex3/+^; mCyp11a1-iCre*	Adenoma, increase adrenal size in male compared to activation of CTNNB1 alone	[41]
	*Ctnnb1^ex3/+^; AS ^Cre/+^*	Hyperaldosteronism, increased rosette frequency in the zG, block differentiation of zG to zF cells	[49,50]
*Dennd1a.V2*	*pCMV-BAM hDenndia.V2* (overexpression of the human V2 isoform)	Overexpression of *Cyp17a1*, phenotype not evaluated	[84]
*Dicer*	*Dicer ^flox/flox^; Nr5a1-Cre* ^high^	Adrenal hypoplasia at e16.5 and adrenal failure at birth	[17,18]
*Ezh2*	*Ezh2 ^flox/flox^; Nr5a1-Cre* ^high^	Aberrant zonal differentiation, loss of PKA activity in the zF, expansion of the zG, appearance of subcapsular spindle-shaped cells, phenotype more pronounced in males	[19]
*Fgfr2*	*Fgfr2 ^flox/flox^;Tbx-Cre*	Adrenal hypoplasia	[10]
	*Fgfr2 ^flox/flox^;Nr5a1-Cre* ^high^	Adrenal hypoplasia	[20]
	*Fgfr2 IIIb ^flox/−^;K5^Cre/+^* (global inactivation via recombination in germ cells)	Adrenal hypoplasia at e15.5	[85]
	*Fgfr2 ^flox/flox^; AS ^Cre/+^*	Impaired rosette formation in the zG	[49]
*Gata4*/ *Gata6*	*Gata4 ^flox/flox^*; *Wt1^CreERt2/+^*	Disruption of coelomic epithelium thickening	[5]
	*Gata4 ^flox/flox^*; *Osr1^eGFP-CreERt2/+^*	Disruption of coelomic epithelium thickening	[5]
	*Gata4 ^flox/flox^*; *Osr1^eGFP-CreERt2/+^*; *Wt1^CreERt2/+^*	Disruption of coelomic epithelium thickening	[5]
	*Gata4 ^flox/flox^; CAG-CreER*	Disruption of coelomic epithelium thickening	[5]
	*Gata4^+/−^*	Reduced subcapsular spindle-shaped cell hyperplasia following gonadectomy	[86]
	*Cyp21a1-Gata4*	Subcapsular spindle-shaped cell hyperplasia	[71]
	*Gata6 ^flox/flox^; Nr5a1-Cre*	Adrenal hypoplasia, absence of an X-zone in postnatal adrenal, Subcapsular spindle-shaped cell hyperplasia	[31]
	*Gata4 ^flox/flox^; Gata6 ^flox/flox^; Nr5a1-Cre* ^high^	Adrenocortical like cells in the testes	[22]
		Adrenal agenesis, Adrenocortical like cells in the testes	[21]
*Gli3*	*Gli3* ^Δ699/ Δ699^	Adrenal aplasia	[87]
		Normal adrenals	[88]
*Hoxb9*	*Nr5a1-Hoxb9*	Large X-zone	[41]
	*Nr5a1-Hoxb9; Ctnnb1^ex3/+^; mCyp11a1-iCre*	Adrenal tumor formation	[41]
*Igf2*	*H19 ^floxDMD/floxDMD^; Nr5a1-Cre^low^*	Normal adrenal	[15]
	*Akr1b7-Igf2*	Subcapsular spindle-shaped cell hyperplasia	[47]
*Insr*/ *Igf1r*	*Insr^−/−^; Igfr1^−/−^* (via recombination of *Insr^flox/flox^; Igfr1^flox/flox^* in germ cells)	Adrenal agenesis and gonadal hypoplasia	[89]
	*Insr^flox/flox^; Igfr1^flox/flox^; hCyp11a1-iCre*	Abnormal hypoplastic adrenal	[39]
*Lats1*/ *Lats2*	*Lats1 ^flox/flox^; Lats2 ^flox/flox^; Nr5a1-Cre*	Transdifferentiation of adrenocortical cells into myofibroblast like cells	[33]
*Lhcgr*	*Lhcgr^−/−^*	Prevention of GATA4 induction and tumor formation in inhα/Tag mice	[70]
*Mc2r*	*Mc2r^−/−^*	Adrenal hypoplasia limited to the zF, zG still present	[90]
*Mst1*/ *Mst2*	*Mst1 ^flox/flox^; Mst2 ^flox/flox^; Nr5a1-Cre*	Premature subcapsular spindle-shaped cell hyperplasia	[34]
*Mrap*	*Mrap^−/−^*	Adrenal hypoplasia limited to the zF (following corticosterone replacement therapy), expansion of WNT/CTNNB1 signaling in the cortex	[91]
*Nr0b1*	*Nr0b1^−/Y^*	Delayed regression of the X-zone	[92]
		Enhanced subcapsular proliferation in young animals followed by progressive adrenal cortex degeneration in male	[93]
	*Nr0b1^flox/Y^; AS ^Cre/+^*	No effect on the differentiation of zG cells into zF cells	[48]
*Nr5a1*	*Nr5a1^−/−^*	Gonadal and adrenal agenesis	[94]
	*Nr5a1* ^+/−^	Adrenal hypoplasia	[95,96]
	*Nr5a1^flox/flox^; mCyp11a1-iCre*	Morphological changes in the shape of steroidogenic cells of the fetal cortex, *Nr5a1-* cells never observed in the definitive cortex	[40]
	*FAdE-Nr5a1*	Hyperplastic adrenal, ectopic thoracic adrenal tissue, incomplete separation of the AP and GP	[97]
	*Nr5a1^2KR/2KR^*	Delayed regression of the X-zone	[92]
		Expansion of SHH+ cells in the zF, presence of *Sox9+* (Sertoli-like cells?) in the cortex, delayed regression of the X-zone	[98]
	*Nr5a1 ^flox/flox^;* AS*^Cre/+^*	Loss of zG (and zF maintenance independent of the zG)	[48]
	*Nr5a1-TR* (overexpression of rat *Nr5a1*)	Subcapsular spindle-shaped cell hyperplasia and nodule formation	[99]
*Osr1*	*Osr1^−/−^*	Gonadal and adrenal agenesis	[66,76]
*Pbx1*	*Pbx1^−/−^*	Adrenal agenesis	[100]
	*Pbx1^+/−^*	Adrenal hypoplasia and smaller X-zone	[101]
*Pde8b*	*Pde8b^−/−^*	Elevated urinary corticosterone	[102]
		Elevated basal serum corticosterone level in female, Subcapsular spindle-shaped cell hyperplasia	[103]
*Pde11a*	*Pde11a-/-*	Persistence or resurgence of the X-zone, higher cAMP levels, higher incidence of subcapsular spindle-shaped cell hyperplasia, milder phenotype in males	[104]
*Porcn*	*Porcn ^flox/flox^; Nr5a1-Cre* ^high^	Normal adrenal	[23]
*Prkar1a*	*Prkar1a ^flox/flox^; Akr1b7-Cre*	Adrenal hyperplasia, increased PKA signaling, hypercorticosteronemia, appearance of subcapsular spindle-shaped cells, resurgence of an X-zone/presumptive zR (origin not evaluated), milder phenotype in males	[26]
	*Prkar1a ^flox/flox^; Nr5a1-Cre* ^high^	Expansion of the zF at the expense of the zG	[25]
		Repress SUMOylation	[24]
	*Prkar1a ^flox/flox^; FadE/Nr5a1-CreERT2*	Normal adrenal (tamoxifen induction at e14.5)	[37]
	*Prkar1a ^flox/flox^; AS ^Cre/+^*	Hypercorticosteronemia, differentiation of lower zF into a presumptive zR, DHEA secretion	[37]
*Rnfr3*	*Rnfr3 ^flox/flox^; Nr5a1-Cre* ^high^	Normal adrenal	[105]
*Rspo3*	*Rspo3 ^flox/flox^; CAG-CreER*	Progressive adrenal cortex degeneration, loss of zG markers	[6]
	*Rspo3 ^flox/flox^; Gli1^CreERt2/+^*	Progressive adrenal cortex degeneration, loss of zG markers	[6]
*Siahi1a*	*Siahi1a^−/−^*	Smaller X-zone and dysregulation of the zG	[106]
*Sfrp2*	*Sfrp2^−/−^*	Ectopic expression of CTNNB1+ cells in the zF	[107]
*Shh*	*Shh ^flox/flox^; Nr5a1-Cre* ^high^	Adrenal hypoplasia (more severe on the right side)	[17,27,28,29]
*Six1*/ *Six4*	*Six1^−/−^; Six4^−/−^*	Potential marginal hypoplastic adrenal gland at 1dpp (unconfirmed, suggested in [107])	[108,109]
*Smo*	*Smo ^flox/flox^; Nr5a1-Cre* ^high^	Normal adrenal	[28]
	Rosa^SmoM2^; *Gli1^CreERt2/+^*	Enhanced subcapsular WNT/CTNNB1 signaling	[53]
*Tcf21*	*Tcf21^LacZ/LacZ^*	Improper separation of the AP and GP	[36]
*Wnt4*	*Wnt4^−/−^*	Reduced aldosterone secretion	[110]
	*Wnt4 ^flox/flox^; Nr5a1-Cre* ^high^	Reduction in zG markers	[6]
		Expansion of the zF at the expense of the zG	[25]
*Wt1*	*Wt1^−/−^*	Gonadal and adrenal agenesis	[61]
	*Wt1^−/−^; WT280* (WT1 complementation)	Rudimentary hypoplastic adrenal gland at e15.5	[77]
	*Cited2^+/−^*; *Wt1^+/−^*	Adrenal hypoplasia	[83]
	*Rosa26^Wt1+KTS/Wt1+KTS^; Nr5a1-Cre* ^high^	Adrenal hypoplasia, subcapsular spindle-shaped cell hyperplasia	[13]
*Yap/Taz*	*Yap ^flox/flox^; Taz ^flox/flox^; Nr5a1-Cre*	Progressive adrenal cortex degeneration in male	[32]
*Znrf3*	*Znrf3 ^flox/flox^; Nr5a1-Cre* ^high^	Adrenal hyperplasia, expansion of the zF, disrupted adrenal organization	[23]
		Development of adrenocortical carcinoma in 78 weeks-old females, activation of androgen-dependent innate antitumor immunity in males	[111]
	*Znrf3 ^flox/flox^; Rnfr3 ^flox/flox^; Nr5a1-Cre* ^high^	Same as the *Znrf3 ^flox/flox^; Nr5a1-Cre*^high^	[23]
	*Znrf3 ^flox/flox^; Porcn ^flox/flox^; Nr5a1-Cre* ^high^	Rescue the phenotype observed in *Znrf3 ^flox/flox^; Nr5a1-Cre*^high^	[23]
	*Znrf3 ^flox/flox^;* AS *^Cre/+^*	Adrenal hyperplasia, expansion of the zF, disrupted adrenal organization, moderate increased WNT/CTNNB1 signaling in the upper zF	[23]

## Data Availability

Not applicable.

## Supplementary Materials

The following supporting information can be downloaded at: https://www.mdpi.com/article/10.3390/ijms232214388/s1.

## Abbreviations

*Abcb1b*	ATP-binding cassette, sub-family B (MDR/TAP), member 1B
*Akr1b7*	aldo-keto reductase family 1, member B7
*Amhr2*	anti-mullerian hormone type 2 receptor
*Apc*	adenomatosis polyposis coli
*Ar*	androgen receptor
*Axin2*	axin2
*Cbx2*	chromobox 2
*Cited2*	Glu/Asp-rich carboxy-terminal domain 2
*Ctnnb1*	beta-catenin
*Cyb5*	cytochrome b5
*Cyp2f2*	cytochrome P450, family 2, subfamily f, polypeptide 2
*Cyp11a1*	cytochrome P450 side chain cleavage enzyme 11a1
*Cyp11b1*	cytochrome P450, family 11, subfamily b, polypeptide 1
*Cyp11b2/As*	cytochrome P450, family 11, subfamily b, polypeptide 2/aldosterone synthase
*Cyp17a1*	cytochrome P450, family 17, subfamily a, polypeptide 1
*Cyp21a1*	cytochrome P450, family 21, subfamily a, polypeptide 1
*Dennd1a*	DENN/MADD domain containing 1A
*Dhcr24*	24-dehydrocholesterol reductase
*Emx2*	empty spiracles homeobox 2
*Ezh2*	zeste homolog 2
*FAdE/Nr5a1*	Fetal adrenal enhancer of nuclear receptor subfamily 5, Group A, member 1
*Foxl2*	forkhead box L2
*Fgfr2*	fibroblast growth factor receptor 2
*Frzb*	Frizzled-related protein
*Gata4*	GATA binding protein 4
*Gata6*	GATA binding protein 6
*Gli1*	GLI-Kruppel family member 1
*Gli2*	GLI-Kruppel family member 2
*Gli3*	GLI-Kruppel family member 3
*Hoxb9*	homeobox B9
IGF	insulin growth factor
*Igf1r*	insulin-like growth factor 1 receptor
*Inha*	inhibin a
*Insr*	insulin receptor
*Lats1*	large tumor suppressor 1
*Lats2*	large tumor suppressor 2
*Lhcgr*	luteinizing hormone/choriogonadotropin receptor
*Lhx9*	LIM homeobox protein 9
MAPK	mitogen-activate kinase protein
*Mc2r*	melanocortin 2 receptor
*Mrap*	melanocortin 2 receptor accessory protein
*Nes*	nestin
*Nr0b1*	nuclear receptor subfamily 0, group B, member 1
*Nr2f2*	nuclear receptor subfamily 2, group F, member 2
*Nr5a1*	nuclear receptor subfamily 5, group A, member 1
*Osr1*	odd-skipped related transcription factor 1
*Pbx1*	pre B cell leukemia homeobox 1
*Pde1b*	phosphodiesterase 1B, Ca2+ calmodulin dependent
*Pde2a*	phosphodiesterase 2A, cGMP-stimulated
*Pde3a*	phosphodiesterase 3A, cGMP-inhibited
*Pde7b*	phosphodiesterase 7B
*Pde8b*	phosphodiesterase 8B
*Pde11a*	phosphodiesterase 11A
PKA	protein kinase A
*Porcn*	porcupine homolog
*Pou5f1*	POU domain, class 5, transcription factor 1
*Prkar1a*	protein kinase, cAMP dependent regulatory, type 1, alpha
*Prep1*	Pbx-knotted 1 homeobox
*Rspo3*	R-spondin 3 homolog
*Shh*	sonic hedgehog
*Siah1a*	seven in absentia 1A
*Six1*	sine oculis-related homeobox-1
*Six4*	sine oculis-related homeobox-4
*Sfrp2*	Secreted frizzled-related protein 2
*Smo*	smoothened homolog
*Taz*	transcriptional co-activator with PDZ-binding motif
*Tcf21*	transcription factor 21
*Tbx18*	T-box18
*Wnt4*	wingless-type MMTV integration site family, member 4
*Wnt2b*	wingless-type MMTV integration site family, member 2
*Wt1*	wilms tumor 1
*Yap*	yes-associated protein
*Znrf3*	zinc and ring finger 3
