# Supplementary material for: Transgenic Mouse Models to Study the Development and Maintenance of the Adrenal Cortex
Source: Int J Mol Sci. 2022 Nov 19;23(22):14388. doi: 10.3390/ijms232214388 (PMC9693478; doi:10.3390/ijms232214388)
Supplement: Supplementary file 1 [file ijms-23-14388-s001.zip › ijms-1977748-supplementary.pdf]

Table S1: Adrenocortical expression and limitations of Cre mouse strains available to study adrenal cortex development and maintenance

Mouse strains	Expression in the adrenal gland	Recombination	Usage	Limitations*
CAG-CreER	- Expression in all cells of the adrenal	- Tamoxifen injection dependent	- Varying (depending on the cell type expressing the gene of interest)	- Timing of tamoxifen injection should be carefully selected<br><br>- Global gene inactivation: Potential indirect effect on the adrenal, might lead to rapid embryonic death if used to study development
Osr1$^{eGFP\text{-}CreERt2}$	- Coelomic Epithelium	- Tamoxifen injection dependent (injection of dams at e8.5)	- Study of coelomic epithelium and early stages of AGP development	- Timing of tamoxifen injection should be carefully selected (*Osr1* is not expressed in the AGP/AP)<br><br>- Variable efficiency, might need to use this strain in combination with the *Wt1*$^{CreERt2/+}$ strain<br><br>- Expressed in intermediate and dorsal lateral plate mesoderms and their descendants (heart, kidney, gonad, muscle connective tissue, fibro-adipogenic progenitors)
Wt1$^{CreERt2/+}$	- Coelomic epithelium/AGP	- Tamoxifen injection dependent (injection of dams at e8.5)	- Study of coelomic epithelium and early stages of AGP development	- Timing of tamoxifen injection should be carefully selected (*Wt1* is not expressed in the AP)<br><br>- Variable efficiency, might need to use this strain in combination with the *Osr1*$^{eGFP\text{-}CreERt2}$ strain to study coelomic epithelium development<br><br>- Expressed in intermediate mesoderm and its descendants (heart, kidney, wolffian duct, gonads…) both in the embryos and postnatally. Also

	- Subpopulation of capsular cells (from e13.5 to adult)	- Injection of dams at e13.5 or postnatally to study capsular cell population	- Tracing *Wt1*+ cell population<br>-study development of subcapsular hyperplastic lesion following gonadectomy	expressed in spleen, breast and smooth muscle<br><br>- *Wt1*+ cells are not the main capsular stem cell population
Gata4^{CreERt2}<br>**(Not characterized except in the digestive tract epithelium, expected results)**	- Coelomic epithelium/AGP	- Tamoxifen injection dependent (injection of dams at e8.5)	- Study of coelomic epithelium and early stages of AGP development	- Timing of tamoxifen injection should be carefully selected (*Gata4* is not expressed in the AP)<br><br>- Potential expression in gonads, neural crest, heart, vasculature, placenta, pancreas, digestive tract (only verified for the digestive tract epithelium)
*Tbx*18^{Cre}	- Anterior coelomic epithelium/ AP	- e10-e10.5	- Study of late AGP and AP development	- Expression in the anterior GP, heart, limb buds, urogenital tract, bladder, inner ear mesenchyme, adipocytes, smooth muscle…<br><br>- Expression in the definitive cortex has not been evaluated
FAdE/Nr5a1-Cre<br><br><br><br><br><br><br><br>*FAdE/Nr5a1-CreERT2*	- Fetal adrenocortical cells and their descendants (adrenocortical cells of the definitive cortex and some capsular stem cells)<br><br>- Fetal adrenal cortex and their descendants forming the definitive cortex	- e10 .5<br>- Tamoxifen injection dependent<br><br><br><br><br><br><br>- Injection of dams between e11.5-e13.5 (recombination in the fetal cortex and definitive cortex)<br><br>- Injection of dams at e14.5 (recombination	- Tracing studies of fetal adrenocortical cells<br><br>- Could potentially be used to study fetal cortex (and definitive cortex) development<br><br><br><br>- Could be used to the fetal cortex (without affecting the definitive cortex)<br><br>- Useful to confirm if a phenotype observed in other models (*Nr5a1-Cre*, *Cyp11b2*^{Cre}) is solely associated with the definitive cortex	- Expression in gonads and some cells in the thoracic region<br><br>-Stochastic recombination in the definitive cortex (percentage of recombined cells not evaluated)<br><br><br><br>- Timing of tamoxifen injection should be carefully selected<br><br>- Expressed in rare gonadal cells<br><br>- Stochastic recombination in the definitive adrenal cortex

		in the fetal cortex only)		
Nr5a1-Cre^{high} (from Keith Parker lab)<br><br>and<br><br>*Nr5a1-Cre* (from Bradford Lowell)<br><br><br><br><br><br><br><br><br><br><br><br><br>*Nr5a1-Cre*^{low} (from Keith Parker lab)<br><br><br><br><br><br>*Nr5a1*$^{eGFP\text{-}CreERt2}$ **(not characterized in the adrenal gland, expected results)**	- AGP/AP, fetal adrenal cortex, definitive adrenal cortex (including subcapsular progenitor cells)<br><br><br><br><br><br><br><br><br><br><br><br><br><br><br>- Same as *Nr5a1*-Cre^{high} but in fewer cells<br><br><br><br><br><br>- Potentially AGP/AP, fetal adrenal cortex, definitive adrenal cortex (including subcapsular progenitor cells)	-e10.5 (exact recombination onset has not been evaluated for the strain from Lowell group)<br><br><br><br><br><br><br><br><br><br><br><br><br><br>-e10.5<br><br><br><br><br><br>- Tamoxifen injection dependent	- Most used models, best characterized models<br><br>- Study of the development of the fetal cortex and development/maintenance/function of the definitive cortex<br><br><br><br><br><br><br><br><br><br><br><br><br>- Interesting to evaluate gain-of-function mutation in proto-oncogene or mutation in tumor suppressor genes (more representative of tumor formation. i.e clonal expansion of few mutated cells)<br><br>- Study of the development of the fetal cortex and development/maintenance/function of the definitive cortex	- Expressed in the gonads (Leydig/Sertoli/Theca/Granulosa), gonadotropes of the pituitary, VMH, subpopulation of dermal fibroblast progenitor, spleen<br><br>- Potential indirect effect of sex hormones due to the inactivation of the targeted genes in testis/ovary/pituitary; Should measure circulating testosterone, oestrogen, LH and FSH levels<br><br>- Inactivation of the targeted gene in the VMH could affect corticosterone/ACTH diurnal feedback.<br><br><br>- Not as useful to study the development of the fetal cortex (recombination in fewer cells)<br><br><br><br><br>- Similar limitations to the postnatal characterization of *Nr5a1*-Cre models
hCyp11a1-iCre<br><br><br><br>*mCyp11a1-iCre*	- Adrenal gland cortex (fetal and definitive), Expression should be limited to steroidogenic cells (expression at	- *hCyp11a1-iCre* (e10.5)	- Study of the development of the fetal cortex and development/maintenance/function of the definitive cortex	- Expressed in fetal and adult Leydig cells, theca cells and corpus luteum of the postnatal ovaries, brain (*hCyp11a1-iCre* and *Cyp11a1*$^{Gfp,Cre/+}$; not evaluated

Cyp11a1$^{Gfp,Cre/+}$	e10.5 in the *hCyp11a1-iCre* suggests it might not be the case)	- *mCyp11a1-iCre* (not evaluated before e14.5)<br><br>- *Cyp11a1*$^{Gfp,Cre/+}$ (not evaluated before e17.5)		in *mCyp11a1-iCre*), fetal ovaries (*hCyp11a1-iCre*)<br><br>- Potential indirect effect of sex hormones due to the inactivation of the targeted genes in testis/ovary. Should measure circulating testosterone, oestrogen, LH and FSH levels.
Akr1b7-Cre	- Steroidogenic cells of the fetal and definitive adrenal cortex	- e14.5 (stochastic recombination, 80% the cells)<br><br>- Not expressed in fetal adrenocortical cells that differentiate into cells of the definitive cortex	- Useful to study function of the definitive adrenal cortex (independently of the fetal cortex)<br><br>- Expression is more specific than *Nr5a1-Cre* and *Cyp11a1-Cre*	- Expressed in the kidney (developing and adult)<br><br>- Recombination less efficient than *Nr5a1*-Cre/ *Cyp11a1-Cre* strains (but more specific)
Cyp11b2^{Cre}	- Steroidogenic zG cells and their descendants (zF cells)	- Rare cells from e16.5 to 1dpp (all zG cells at 6 weeks)<br><br>- Recombination in all zF cells at 3 months in females and approximately 9 months in male	- Best model to study zG morphogenesis and functions (no recombination in fetal/definitive cortex before zG formation)<br><br>- Useful to study zG to zF centripetal transdifferentiation/adrenal maintenance and perform tracing studies	-Mosaic of recombined and not recombined zG cells (rapid transdifferentiation of stem and progenitor cells population to restore zG functions)<br><br>- zF cell maintenance can become independent of zG cell transdifferentiation in some models
Cyp11b1$^{eGFP\text{-}Cre}$ **(not fully characterized)**	- zF cells (double marking/higher magnification images needed to determine if some zG cells are recombined or not)	- Unknown (not evaluated in the embryo)	- Potentially useful to study maintenance/functions of the zF.	- Model not well characterized (expression in the fetal adrenal cortex, and potential expression in the fetal gonads not evaluated), only one article published in Chinese
Gli1^{CreERt2}	- Subpopulation of capsular (stem cells) (and their descendants)	- Tamoxifen injection dependent	- Useful to target capsular stem cells<br><br>- Study of adrenal cortex maintenance	- Expressed in kidney, brain, astrocytes, fibroblast, chondrocytes, adipocytes, hair follicle, prostate, perisoteal cells, Leydig cell, theca cells…

			- Tracing experiment to study centripetal transdifferentiation	- Capsular stem cells do not normally contribute to adrenal maintenance in adult males<br><br>- Potential indirect effect of sex hormones due to inactivation of the targeted genes in Leydig and theca cells. Should measure circulating testosterone, oestrogen, LH and FSH levels
Shh^{Cre}<br><br>*Shh*^{CreERt2}	- Cortex periphery in the forming definitive cortex, subcapsular progenitor cells of the zG (and their descendants)<br><br>- Subcapsular progenitor cells of the definitive cortex in the zG (and their descendants)	-e11.5<br><br>-Tamoxifen injection dependent	-Tracing experiment of progenitor cells<br><br>- Mostly used for tracing experiment of progenitor cells	- Expressed in developing limb buds, heart, ventral neural tube, lung epithelium, stomach endoderm, intestinal epithelium, external genitalia, neurons, prostate, secondary heart field…<br><br>- Expressed in bladder epithelial stem cells, papillary collecting duct, uritheric epithelium, hair follicle, brain astrocytes, epithelial taste bud progenitors, spleen, gastric parietal cells…
Axin2^{CreERt2}<br><br>and<br><br>*Wnt4*^{CreERt2}	- Cortex periphery in the forming definitive cortex (and their descendants)<br><br>- Postnatally, Adrenal zG cells (including the subcapsular progenitors and their descendants)	- Tamoxifen injection dependent	- Mostly used for tracing experiment of zG cells (including subcapsular progenitors)	- Expressed in intestinal epithelium stem cells, mammary gland, neural stem cells, ovaries, liver, kidney… (*Axin2*)<br><br>-Expressed in kidney, oesophagus, ovary, skin, small intestine… (*Wnt4*)
Nes-CreERt2	- Stress induced progenitor cell populations of	- Tamoxifen injection dependent	- Tracing and purification of stress-induced progenitor cells	- Expressed in glial progenitor cells, astrocytes, oligodendrocytes

	steroidogenic cells of the adrenal cortex and of chromaffin cells (and their descendants)		- Evaluation of stress adaptation	- Should consider the contribution of other adrenocortical stem/progenitor cells to stress response

* Postnatal phenotypes should be evaluated in males and females as the adrenal cortex is sexually dimorphic
